# Recent Advances in Biopolymer-Based Hydrogel Electrolytes for Flexible Supercapacitors

**DOI:** 10.1021/acsenergylett.3c02567

**Published:** 2024-03-29

**Authors:** Jiansen Ding, Yang Yang, Jade Poisson, Yuan He, Hua Zhang, Ying Zhang, Yulan Bao, Shuiliang Chen, Yong Mei Chen, Kai Zhang

**Affiliations:** † College of Bioresources Chemical and Materials Engineering, National Demonstration Center for Experimental Light Chemistry Engineering Education, Shaanxi University of Science & Technology, Xi’an 710021, P. R. China; ‡ State Key Laboratory of Pulp and Paper Engineering, South China University of Technology, Guangzhou 510640, P. R. China; § College of Chemistry and Chemical Engineering, Jiangxi Normal University, Nanchang 330022, P. R. China; ∥ Sustainable Materials and Chemistry, University of Göttingen, Büsgenweg 4, 37077 Göttingen, Germany

## Abstract

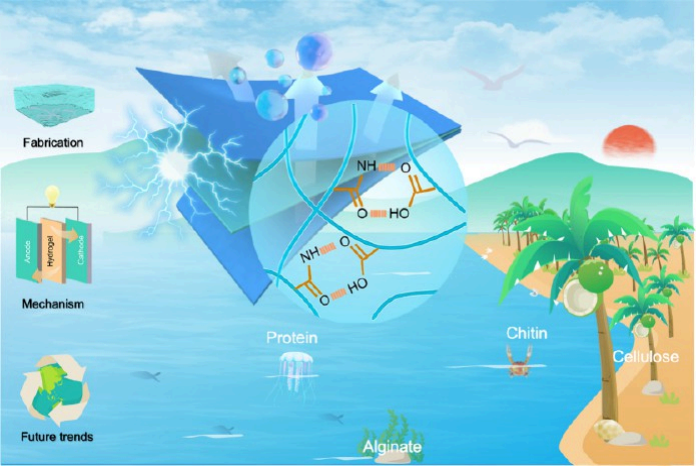

Growing concern regarding the impact of fossil fuels has led to demands for the development of green and renewable materials for advanced electrochemical energy storage devices. Biopolymers with unique hierarchical structures and physicochemical properties, serving as an appealing platform for the advancement of sustainable energy, have found widespread application in the gel electrolytes of supercapacitors. In this Review, we outline the structure and characteristics of various biopolymers, discuss the proposed mechanisms and assess the evaluation metrics of gel electrolytes in supercapacitor devices, and further analyze the roles of biopolymer materials in this context. The state-of-the-art electrochemical performance of biopolymer-based hydrogel electrolytes for supercapacitors and their multiple functionalities are summarized, while underscoring the current technical challenges and potential solutions. This Review is intended to offer a thorough overview of recent developments in biopolymer-based hydrogel electrolytes, highlighting research concerning green and sustainable energy storage devices and potential avenues for further development.

Given the rapid progress in fields such as electric vehicles and smart grids, the importance of highly efficient energy storage systems for sustainable energy development and energy security cannot be overstated. Supercapacitors have emerged as promising energy storage candidates, demonstrating obvious advantages such as outstanding lifespan (>100,000 cycles), excellent safety, and a wide temperature operating range (−50 to 200 °C).^1^ Compared to conventional lithium-ion batteries, they enable remarkably rapid charging–discharging rates (within seconds to minutes) and superior power density (>10 kW kg^–1^).^2^ Flexible supercapacitors, featuring pliable electrodes and electrolytes, are particularly suitable for powering lightweight wearable electronics. However, in practical applications, flexible supercapacitors are more susceptible to various deformations under mechanical strains, especially bending and shearing forces. This limitation has stimulated innovation in electrode, electrolyte, separator, current collector materials, and interface bonding techniques to mitigate mechanical mismatch while preserving the flexible supercapacitors’ excellent electrochemical performance.^3^

The electrolyte serves as the indispensable ionic conductor between the two electrodes in supercapacitors, exerting a significant influence on the electrochemical performance with regard to various aspects, including the electrochemically stable potential window, specific capacity, power density, energy density, cycle stability, and safety.^4,5^ Gel polymer electrolytes (GPEs), which contain immobilized liquid electrolytes in a polymer matrix, have been proposed to simultaneously act as both separator and electrolyte, reducing the risk of leakage and evaporation observed in devices using liquid electrolytes and the rigidity of solid electrolytes.

Up to now, various synthetic polymers, such as polyethylene,^6^ polypropylene,^7^ and polyacrylonitrile,^8^ have been used extensively as polymer matrices for GPEs. Compared with synthetic polymers, biopolymers, such as polysaccharides (e.g., cellulose, alginate, chitin/chitosan) and protein-based polymers (e.g., silk, gelatin), have attracted attention due to their natural abundance, low cost, biodegradability, good biocompatibility, and sustainability. Moreover, their tunable morphology and mechanical properties, along with abundant reactive sites amenable to chemical modification, render them as appealing building blocks for sustainable electronic devices. Their abundant functional groups provide an opportunity for the efficient migration of various metal cations.^9^ In addition, nanocellulose materials, e.g., nanofibrils, cellulose nanocrystals, and bacterial cellulose, demonstrate high mechanical strength, structural flexibility, and tunable self-assembly behavior. Consequently, extensive endeavors have been undertaken to fabricate multifunctional GPEs from biopolymer-based gel matrices.

While some reviews have touched upon the utilization of biopolymer-based GPEs in flexible supercapacitors, they rarely delve into the intricacies of incorporating biopolymers into gel electrolytes for supercapacitors, nor have they proposed effective strategies to enhance the performance of such devices.^9−12^ In this Review, we aim to comprehensively discuss recent advances of biopolymer-based GPEs and their applications in energy storage devices, especially supercapacitors. First, representative examples of biopolymers and their corresponding structures and properties are briefly introduced, and their applicability as GPEs in flexible supercapacitors is described. Next, we describe the energy storage mechanisms of supercapacitors, revealing the pivotal roles and functions that biopolymers play in facilitating ion migration within GPEs. Additionally, the performance evaluation standards of biopolymer-based GPEs for flexible supercapacitors are highlighted. Furthermore, detailed insights into various strategies that have been explored to enhance the performance of biopolymer-based GPEs are provided. Such strategies include methods to optimize interface contact and voltage windows. Finally, we propose and summarize the current challenges and future prospects of biopolymer-based GPEs for flexible supercapacitors.

## Structure and Properties of Typical Biopolymers and Their Roles in Electrolytes

**Figure 1:**
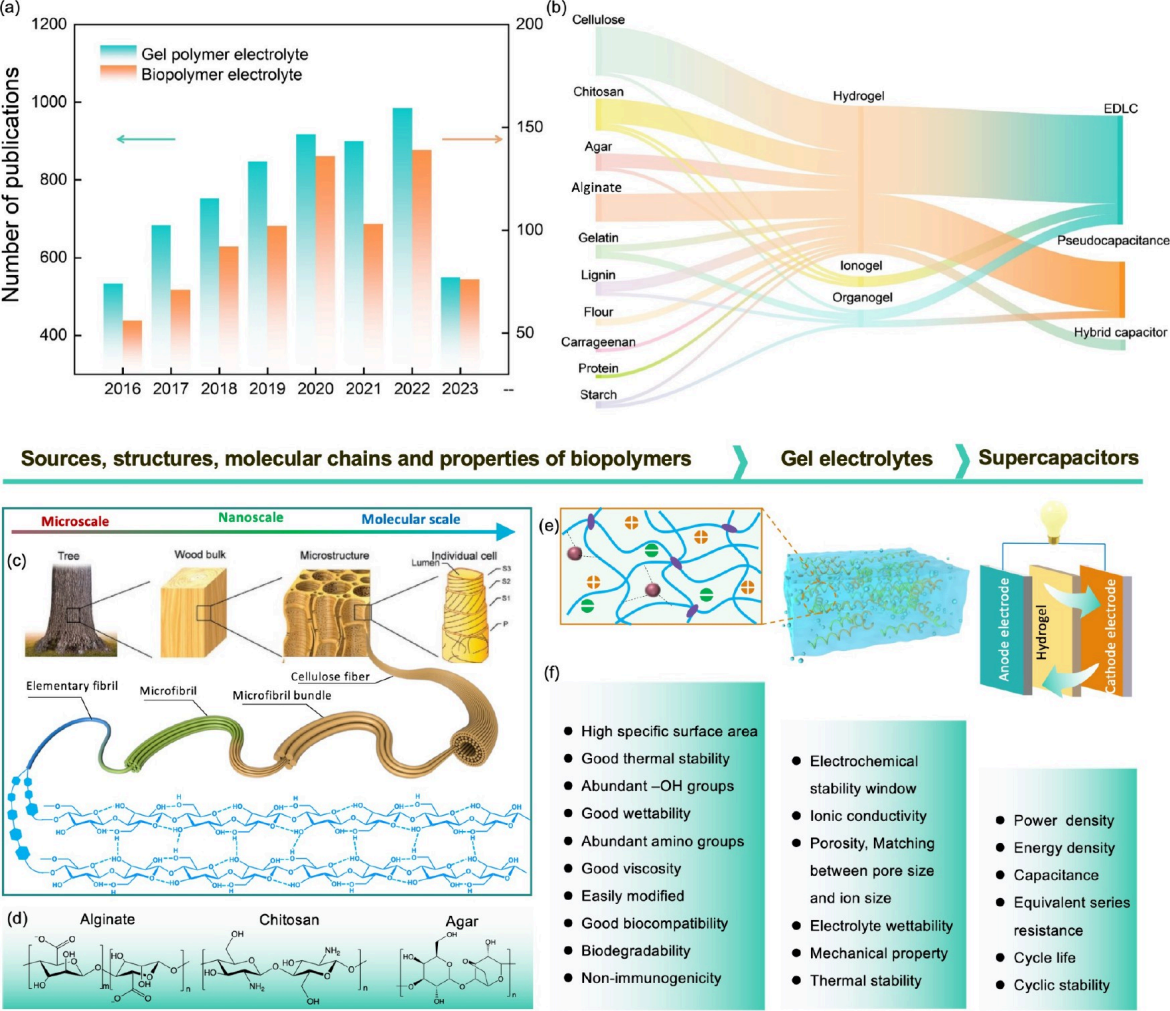
Hierarchical structure, molecular structures, and features of the biopolymers most commonly used for fabricating the various hydrogel electrolytes for supercapacitors. (a) Numbers of publications devoted to biopolymers-based gel electrolytes for supercapacitors within the past few years. (b) Visualization of different biopolymer-based gel electrolytes and their use in various types of supercapacitors. (c) Hierarchical cellular structure of wood with pronounced anisotropy, in which the cell wall is composed of microfibril bundles that can be divided into microfibrils, nanofibrils, elemental fibrils, and molecular chains at different length scales. Reproduced with permission from ref (15). Copyright 2021 Wiley-VCH. (d) Molecular structures of alginate, chitosan, and agar. (e) Schematic illustration of a biopolymer-based hydrogel electrolyte and its application in supercapacitors. (f) Typical features of biopolymers and the performance requirements of the corresponding hydrogel electrolyte for supercapacitors.

Biopolymer-based GPEs have been used extensively in supercapacitor applications in recent years (Figure 1a). Among them, cellulose is the dominant raw material for the preparation of GPEs, followed by chitosan, gelatin, and alginate. These versatile materials are employed as additives or polymer network frameworks to develop diverse hydrogels, ionogels, and organogels, which find considerable utility across various types of supercapacitors (Figure 1b). The abundant functional groups prevalent in biopolymers can be harnessed, thereby facilitating the realization of efficient energy storage strategies in synergy with sustainable energy devices.

### Typical Biopolymers

Cellulose, derived from various plants (e.g., wood, bamboo, cotton, agricultural crops), bacteria, and marine algae, is the most abundant natural biopolymer in the world. It consists of linear β-1,4-linked d-glucose units and has abundant hydroxyl groups (−OH) that can form inter- and intramolecular bonds between polymer chains.^13^ Therefore, these strong hydrogen-bond networks endow cellulose with high stability and axial stiffness. In plants, the intermolecular hydrogen bonds and van der Waals interactions between neighboring glucose molecules contribute to the parallel stacking of macromolecular cellulose chains, which assemble into elementary fibrils with widths of ∼3–5 nm and lengths of over several hundred nanometers. Tens of these elementary fibrils then assemble into rectangular arrays surrounded by hemicelluloses and lignin, forming microfibrils with widths of ∼10–30 nm.^14^ These microfibrils hierarchically pack together, forming the cell walls of the plant (Figure 1c).

From a top-down perspective, micron-sized cellulose fibers can be obtained by removing lignin and hemicelluloses from plants. These cellulose fibers can be further divided into highly ordered and disordered structures.^16^ Cellulose is often considered sparingly soluble in many common solvents due to a high degree of crystallinity imparted by rich hydrogen bonding. This limits the processability of native cellulose into functional materials. By means of top-down methods (e.g., acid hydrolysis and mechanical exfoliation of natural cellulose), cellulose fibers can form various morphologies, such as nanofibrils and cellulose nanocrystals.^17^ Cellulose can also be dissolved and regenerated through exposure to an alkali hydroxide–urea system, ionic liquids, and other green solvents, followed by a phase-conversion process through immersion in a water bath.^18^ Furthermore, bacterial cellulose can also be biosynthesized from cellulose via a bottom-up method.^19^

As a result, the hierarchical nature endows cellulose with unique features that enable them to be used as functional building blocks for various applications, especially in flexible electronics. Examples of such features include the following:^20^ (1) Dielectric and piezoelectric properties make them competitive candidates for important structural components. (2) Abundant hydroxyl groups give cellulose a high degree of functionalization potential. (3) Rich inter- and intramolecular hydrogen bonds endow cellulose with mechanical properties favorable for flexible and stable supercapacitors. In particular, nanocellulose often has a high Young’s modulus of over 100 GPa and estimated strength of several GPa, which can be utilized to develop self-standing and high-strength materials. (4) Activated carbon materials often possess high specific surface areas and rich porous microstructures achieved through a carbonization process. (5) The high aspect ratio of cellulose can be harnessed to form entangled network structures with controlled porous microstructures. Moreover, good wettability and thermal stability make cellulosic materials attractive as separators or gel electrolytes to facilitate ionic transportation.^21^ Given the numerous advantageous characteristics of cellulose, a variety of cellulose-based functional materials, including fibers,^18^ films,^19^ papers,^22^ and hydrogels,^23^ have been developed for flexible supercapacitors, including substrates, electrochemical electrodes, separators, and electrolytes.^24^

Biopolymers with different functional groups (Figure 1d), such as chitosan,^10^ alginates,^10^ lignin,^25^ agar,^26^ gelatin,^27^ and so on, are also potential candidates for the development of a high-performance electrolyte. Each of them possesses similar or identical characteristics that endow the hydrogels with unique performance improvements, which leads to significant enhancement in the overall performances of supercapacitors when combined with suitable electrode assembly (Figure 1e,f).

### Roles of Biopolymers

**Figure 2:**
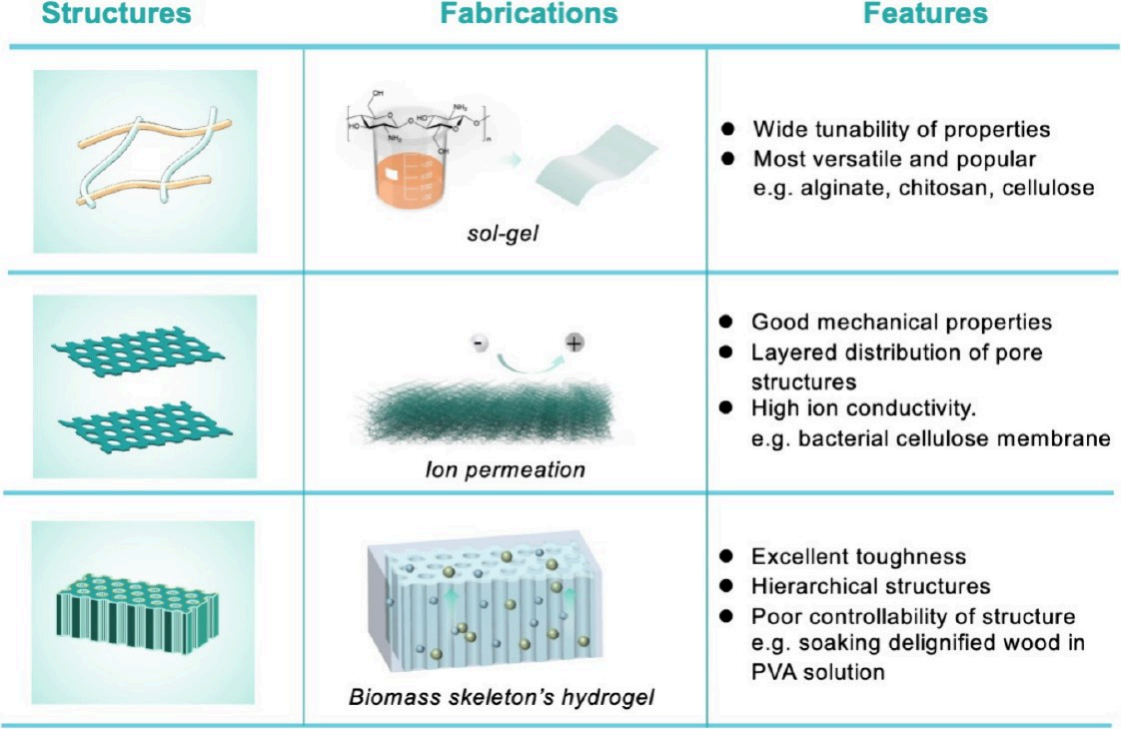
Three main categories of gel electrolytes as well as their fabrication process and representative characteristics, where biopolymers play roles as matrices, additives, and skeletons, respectively.

The gel electrolyte is a 3D flexible material obtained by combining a cross-linked polymer backbone that has been swollen in an appropriate solvent with conductive materials.^10^ There are three major strategies for creating gels employing biopolymers as raw materials, namely, biopolymer materials as matrices or additives, and as skeletons to enhance the performance of gels (Figure 2).

Natural biopolymers can be used directly as an electrolyte matrix through a simple sol–gel strategy in an inorganic salt system, ionic liquid, or urea/NaOH system.^28^ For example, cellulose powders dissolved in an aqueous solution containing ZnCl_2_/CaCl_2_ under high temperatures can be used directly as the hydrogel electrolyte.^29^ However, in most cases, the processing of a biopolymer for practical applications in supercapacitors is limited owing to its highly ordered structure and strong inter- and intramolecular hydrogen bonding. Furthermore, biopolymer-based hydrogels often suffer from weak mechanical properties despite their high versatility. Currently, the most prevalent approach is incorporating biopolymers as an additive with synthetic polymers or other materials. These methods enable the fabrication of homogeneous ion/polymer blends that can establish a continuous ion mobility pathway. For example, when biopolymers are employed as additives for synthetic polymers (e.g., poly(vinyl alcohol) (PVA)), the kinetics and degree of polymerization of the amorphous polymer segments are easily affected by entanglement of molecular chains, modulating the mechanical behavior and conductivity of the GPEs.^30^ The interactions between surface functional groups in biopolymers, as well as their compatibility and dispersion within other components, greatly affect the overall performances of the devices. Surface modification procedures, such as the functionalization of carboxyl and amino groups, were developed to improve the compatibility of solvents and other materials with biopolymers.^31−33^ As a typical example, chitosan can be endowed with amphiphilic properties by adjusting the degree of carboxylation.^34^ The results demonstrated that the strong ion–dipole interaction plays a dual role of both retaining water molecules more tightly and optimizing the ion migration pathway within the polymer matrix. In addition, to improve the interfacial connection between biopolymer-based materials and the hydrogel matrix, plasticizers (e.g., glycerol and glutaraldehyde, tannins) can be added to improve the mechanical properties of biopolymer-based hydrogels. For example, Chen et al.^35^ described the preparation of an organogel employing PVA/calcium alginate as the substrate and three alcohols (ethylene glycol, glycerol, and sorbitol) as bridging molecules. The organogel displayed high mechanical properties with a maximum stress of 2.4 MPa, due to the extensive hydrogen bonding between the components.

In addition, gel electrolytes obtained by building porous skeleton networks with biopolymer-based materials have also attracted widespread attention. The primary advantage of employing biopolymers as a framework lies in the ability to optimize their mechanical properties without compromising their electrochemical performance. For instance, a gel electrolyte prepared by filling a bacterial cellulose scaffold with a gel matrix exhibits high mechanical stress of 1.58 MPa and nearly 50% enhancement in the original electrical conductivity of 1.24 S m^–1^.^36^ The surface hydroxyl groups on the bacterial cellulose framework can attract counterions and provide additional hopping sites for ion transfer. Besides, a gel electrolyte with a high electrolyte uptake of 1100 wt% was prepared by immersing a self-supporting porous lignocellulose membrane in a salt solution.^37^ It exhibits good flexibility and an ionic conductivity similar to that of the liquid electrolyte. Moreover, the delignified wood can also be used as a gel scaffold to control the swelling behavior and enhance the mechanical properties of the hydrogels in salt solutions. Its porous structure also facilitates the transport of electrolyte ions.^38^ Similarly, Gao et al.^39^ combined quaternized gelatin with poly(acrylic acid-*co*-acrylamide) gelled *in situ* on a flexible wood scaffold. The good synergy of a hydrophilic quaternary ammonium group and the wood skeleton endows the flexible wood-based hydrogel with an ionic conductivity as high as 5.57 × 10^–2^ S cm^–1^. A porous framework can indeed optimize the mechanical properties of the hydrogel and enhance its capacity for encapsulating liquid electrolytes. However, the utilization of bulky biomass materials for the framework reduces the overall energy density of the device, while also introducing challenges such as an uneven distribution of fibers and pore structures. The dimensions of the pores influence the surface charge density and potential distribution, playing a critical role in the ion transport behavior within electrolyte systems. Thus, the creation of ultrathin and pore-controllable biomass frameworks remains a challenging task to be addressed in future research.

## Energy Storage Mechanism of Supercapacitors

Supercapacitors, also known as electrochemical capacitors, are mainly composed of electrodes (cathode and anode) and electrolytes (organic, ionic, or aqueous). They can be divided into two main types, electric double-layer capacitors (EDLCs) and pseudocapacitors, according to the nature of the interaction mechanism between the electrode–electrolyte interface and the materials themselves.^10^ As a type of physical energy storage, EDLCs rely solely on electrostatic adsorption, with no redox reactions occurring at the electrode–electrolyte interface.^40^ Conversely, pseudocapacitors store the charge through a reversible Faraday redox reaction occurring on the surface of the electrode material, as well as inside the electrode.^41^ These two types can be simultaneously combined into hybrid asymmetric supercapacitors by assembling the battery-type Faraday electrodes as energy sources and the capacitive electrodes as power sources, enabling higher electrochemical energy storage performances than either type alone.^42^

### Energy Storage Mechanism of an Electrical Double-Layer Capacitor

**Figure 3:**
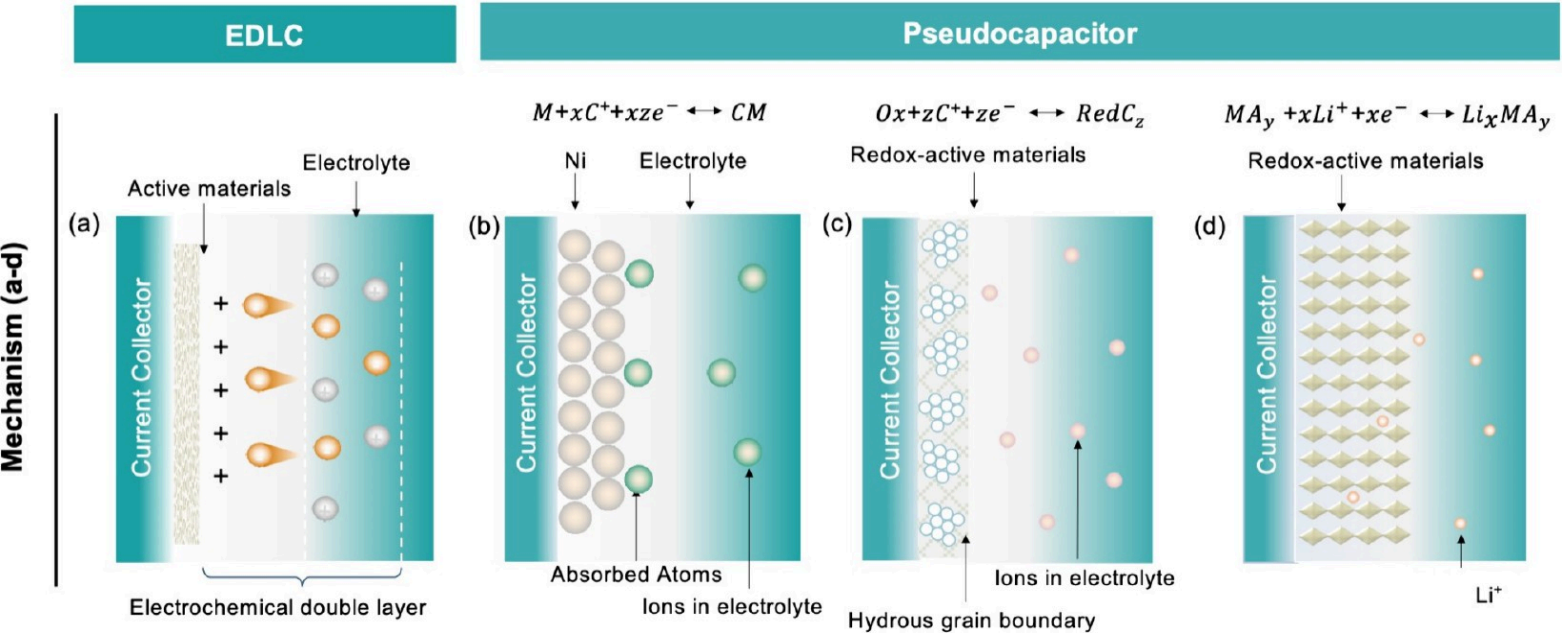
Schematic ion storage mechanisms and electrochemistry of the EDLC reaction and pseudocapacitive processes. (a) The energy storage mechanism of EDLC. (b–d) Different types of reversible redox mechanisms that give rise to pseudocapacitance: (left) underpotential deposition, (center) redox pseudocapacitance, (right) intercalation pseudocapacitance. Reproduced with permission from ref (42). Copyright 2023 Wiley-VCH. (*C* is the atom adsorbed on the surface of the electrode material. *M* is a metal electrode. *z* is the valence state of the adsorbed atom, *x* is the number of adsorbed atoms, so *xz* is the number of transferred electrons. *Ox* is the pseudocapacitive oxide and *Red* is the reduced state of the pseudocapacitive oxide. *MA*_*y*_ is a layer-lattice interpolated host material.)

The EDLC, first proposed by Helmholtz in 1853, is a device that accumulates electrostatic charge in the electrode–electrolyte gap to generate capacitance.^43^ As shown in Figure 3a, the anions and cations in the electrolyte move toward the positive and negative poles, respectively, under the exertion of an electric field, forming opposite charges at the electrolyte–electrode interface to balance the internal electric field of the solution. This design consequently develops electrochemical double-layer capacitance.

The capacitance of EDLCs can be expressed by eq 1:
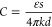
1where ε is the dielectric constant of the electrolyte, *s* is the specific surface area of electrode material, *k* is the electrostatic force constant, and *d* is the effective thickness of the electric double layer.

**Figure 4:**
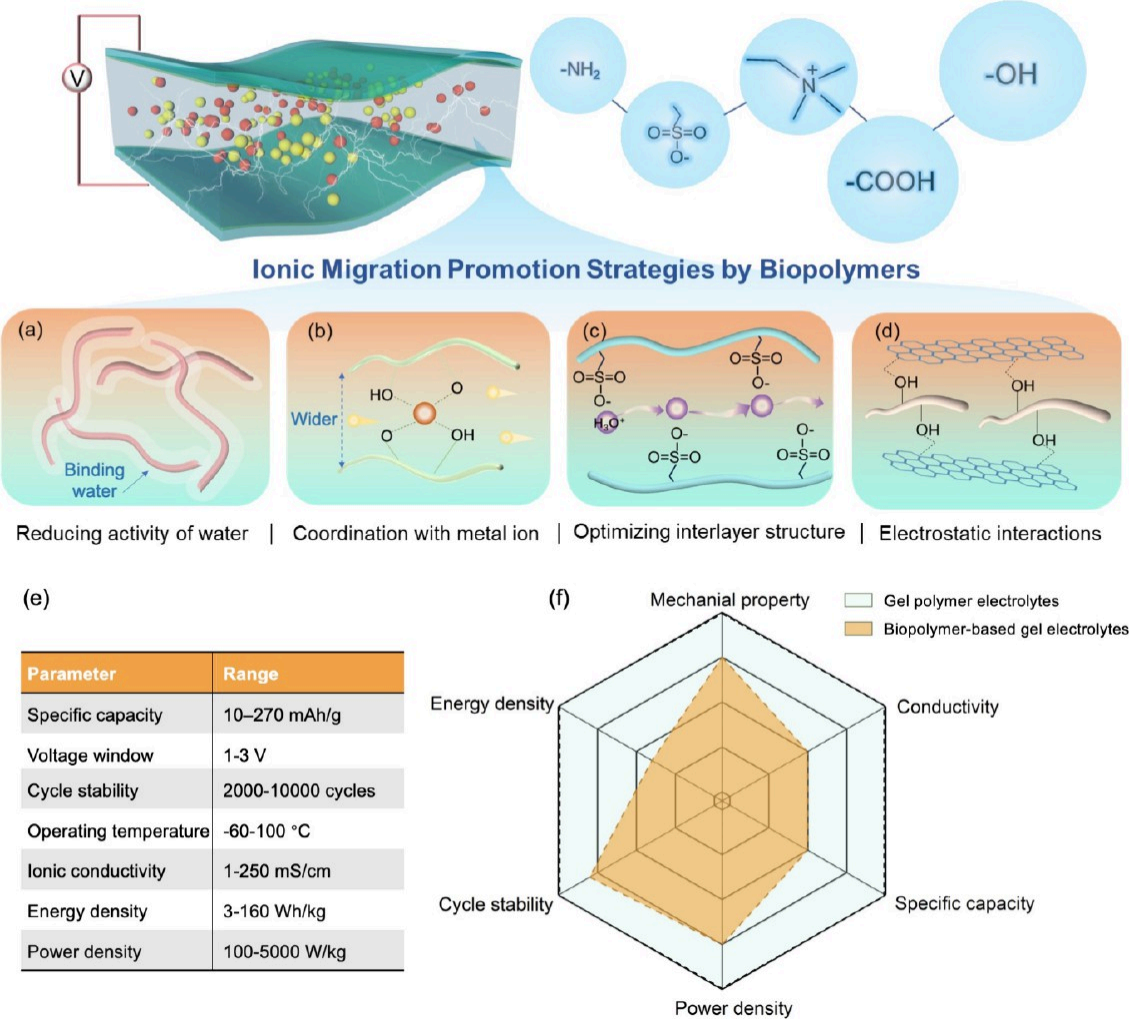
Schematic of representative ionic migration promotion strategies using biopolymers. (a) A single zwitterionic polymer network is bundled together to form multiple interface-wetting water channels, promoting ion migration along the polymer chain. Reproduced with permission from ref (46). Copyright 2021 Elsevier. (b) Coordination of ions (Ca^2+^) with cellulose molecular chain broadens the molecular channels, enabling the inserting and transporting of ions along polymer chains. Reproduced with permission from ref (51). Copyright 2022 American Chemical Society. (c) Sulfonic groups on cellulose chains with negative charges promote proton migration through electrostatic interactions. Reproduced with permission under a Creative Commons CC-BY4.0 license from ref (53). Copyright 2022 Wiley-VCH. (d) Agglomerated two-dimensional nanosheets dispersed along cellulose chains. (e) Table summarizing the current performance parameters of biopolymer-based gel electrolytes. (f) Comparison of the performances of biopolymer-based gel electrolytes with conventional gel polymer electrolytes.

Upon removing the electric field, equal and opposite charges will be generated on both positive and negative electrodes to maintain the stability of the entire system by balancing the potential difference in the electrolyte. By alternatively applying and removing the electric field, the current responds via the rapid movement of the ions in the electrolyte to keep the system electrically neutral, which is the so-called charge–discharge principle of EDLCs.^44^ Therefore, EDLCs enable the storage and release of energy and accumulation of charge through electrostatic adsorption/desorption on the surface of the electrode materials. Unfortunately, the specific capacities of EDLC devices are generally low because the charge storage occurs only on the surface of the electrode material, resulting in a low energy density (∼5 Wh kg^–1^) but high power density (>10 kW kg^–1^).^45^ Since the voltage is directly correlated to energy density, numerous efforts have been devoted to broadening the electrolyte electrochemical window to enhance the energy density of EDLC. The most common strategies involve the introduction of non-aqueous electrolytes including organic and ionic electrolytes,^45^ because the organic electrolyte-based and ionic liquid-based supercapacitors generally have larger potential windows of about 2.5–2.7 and 3.5–4.0 V, respectively. Additionally, another potential method is to reduce the activity of water to widen the voltage window of aqueous electrolytes. Based on the biocompatible sulfobetaine monomers, a hydrogel electrolyte containing an aqueous solution of LiTFSI at a low concentration is proposed as an EDLC device,^46^ which is a fascinating strategy to widen the voltage window (Figure 4a). The amphiphilic zwitterionic polymers (i.e., sulfobetaine) contain both cationic and anionic charging groups, which have a strong water retention capacity and thus promote ionic migration across their structure. Biopolymers possess various hydrophilic functional groups, exhibiting excellent water-binding capabilities. However, there is still a lack of strategies to restrict water activity to enhance the voltage window of aqueous electrolytes. The key challenge lies in controlling the pore size and surface properties of polymers by introducing different hydrophilic or hydrophobic groups, which can influence the affinity and surface tension of water molecules.

### Energy Storage Mechanism of a Pseudocapacitor

The pseudocapacitor, which is also known as a Faraday capacitor, was originally defined by Conway to describe materials that exhibit similar electrochemical characteristics to conventional capacitors (EDLCs) but involve different charge storage mechanisms.^47^ According to the capacitive electrochemical mechanisms, these are divided into the following three types:

(a) Underpotential deposition (Figure 3b), in which a metal electrochemically deposits onto the surface of another material when the working voltage exceeds the equilibrium potential, producing a metal current collector with a nanostructure. However, the redox potential of a pseudocapacitor is typically small ranging from 0.3 to 0.6 V, and thus the capacitance value is affected by the underpotential deposition process, which restricts the energy density compared to other pseudocapacitor types.^48^ Typically, the interaction of metal ions with hydrophilic groups (e.g., amino, hydroxyl, and/or carboxyl groups) alters the original hydrogen-bonding network, which leads to an erratic deposition process, resulting in reduced stability.
(b) Oxidative reduction (Figure 3c), which is a pseudocapacitor energy storage method dominated by redox reactions. Charge is stored primarily through electron transfer generated by rapid, reversible Faraday redox reactions on or near the surface of electrode materials (i.e., transition metal oxides, and conductive polymers).^42^ Raravikar et. al^49^ compared the pseudocapacitive behavior of several biocompatible hydrogel electrolytes assembled with transition metal electrodes. The oxidation–reduction reactions in hydrogels primarily rely on the Lewis acid–base interactions between the amine and hydroxyl side groups distributed along the polymer chain. Additionally, gels containing amine groups are capable of storing more energy than those composed solely of hydroxyl functional groups. In other words, the effective usage of functional groups in biopolymer materials contributes to an increase in the total energy exchange of the redox reaction.
(c) Intercalated pseudocapacitors (Figure 3d), which mainly store energy by smoothly embedding/detaching electrolyte ions (K^+^, Na^+^, Li^+^, etc.) inside the tunneled or layered electrode material, without an accompanying crystal structure transition during the redox reactions.^47^ Limited by the kinetics of ion diffusion and electron transport in the electrolyte phase, the intercalation process of ions on the electrode surface is slow.^50^ Wang et al.^51^ demonstrated that bacterial cellulose coordinated with calcium ions enabled rapid ion insertion and transport (Figure 4b). Through the coordination interactions, the distance between the cellulose molecular chains is widened, so the ions can move fast along the polymer chains.

Since pseudocapacitance occurs both on the surface and inside the electrode, the specific capacitance and energy density of pseudocapacitors are orders of magnitude higher than those of EDLCs. However, compared with rechargeable batteries, a pseudocapacitor’s energy density is poor as an instantaneous power supply, limiting its application.^41^ The incorporation of multipurpose functional groups in biopolymers can modify the charge-transfer properties of electrolytes, regulate the surface activity of electrolytes, and control the reaction rate at the electrode–electrolyte interface, thus influencing the energy density and power density of energy storage devices. Therefore, it is likely that the modulation of surface energy of adsorption sites can effectively stabilize interfacial electrochemistry.^52^ In the sodium alginate (SA) chain, the negatively charged COO^–^ groups can interact with solvated metal ions. This interaction can modulate the solvation structure, promote migration behavior, and thus facilitate effective ion transfer and reversible deposition/stripping.

## Performance Evaluation Standards of Hydrogel Electrolytes for Supercapacitors

The properties of the gel electrolyte greatly impact the performances of supercapacitors regarding their energy density, power density, capacitance, cycle life, etc. Therefore, the design and fabrication of gel electrolytes with appropriate structures and functions are critical to obtaining high-performance supercapacitors.^54^ Herein, we will analyze the key factors of the gel electrolyte on the performances of supercapacitors from the perspective of basic electrochemical parameters, and further analyze the performances of the biopolymer-based gel electrolytes applied to multifunctional supercapacitors. The properties of the internal structure were also explored to elucidate its correlation to the overall performances of the gel electrolytes.

### Basic Electrochemical Performance of Supercapacitors

To characterize the electrochemical performance of supercapacitors, ionic conductivity, electrochemical impedance spectroscopy (EIS), cyclic voltammetry (CV), and galvanostatic (constant current) charge–discharge (GCD) cycling are typically employed.^55^ Such techniques can be used to measure two key indicators, namely capacitance and resistance. Subsequently, other parameters such as energy content, power capability, round-trip efficiency (Coulombic efficiency and energy efficiency), and cycle life can also be determined.^56^ Each of these techniques is particularly useful for determining a subset of these parameters. For example, EIS is more suitable to measure equivalent series resistance, while GCD curves are more often used to determine the capacitance, energy, and power of supercapacitors.

**Figure 5:**
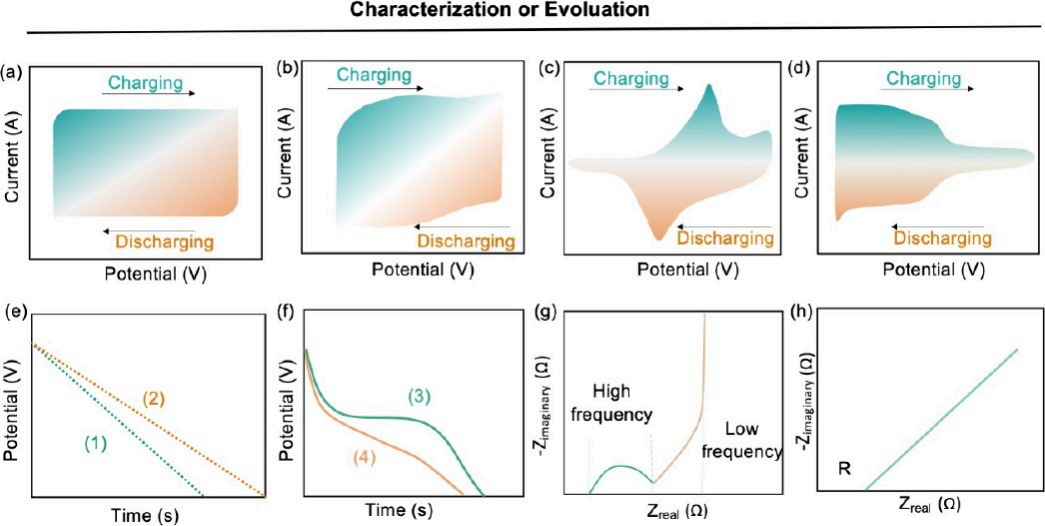
Corresponding CV curves of EDLC and three types of pseudocapacitors: (a) EDLC, (b) underpotential deposition, (c) redox pseudocapacitance, and (d) intercalation pseudocapacitance. Reproduced with permission from ref (45). Copyright 2022 Elsevier. (e, f) Schematic representation of GCD curves for EDLCs and pseudocapacitors: (1) EDLC, (2) underpotential deposition, (3) redox pseudocapacitance, and (4) intercalation pseudocapacitance. Reproduced with permission from ref (56). Copyright 2019 The Royal Society of Chemistry. (g, h) Schematic representation of Nyquist plots for EDLC devices and hydrogel electrolytes, respectively. Reproduced with permission from ref (57). Copyright 2021 Wiley-VCH.

CV is an effective technique to elucidate the reversibility and kinetics of charge-transfer processes. CV involves the monitoring of the current in response to a potential bias that is modulated linearly with respect to time.^57^ For ideal EDLCs, the CV curve resembles a rectangle indicating that no redox reactions are taking place in the electrochemically stable potential window (Figure 5a). The redox reactions occurring in pseudocapacitors can be limited to the surface or near-surface volume of the material, resulting in a rectangular CV curve similar to that of the EDLC (Figure 5b).^58^ However, pseudocapacitive materials can also be characterized by widely distributed charge-transfer peaks that mirror each other during cathodic and anodic scanning,^59^ leading to a pair of distinct redox peaks corresponding to the deposition/dissolution of metal oxides (Figure 5c).^60^ Moreover, intercalated pseudocapacitor materials exhibit highly reversible oxidation state changes during the charge–discharge process, characterized by CVs with significantly broadened peaks (Figure 5d).

The electrochemical stability of the capacitor can be assessed by performing multiple cycles of CV. Moreover, the capacitance can be ascertained by integration of the voltammogram, which is expressed using the following equation:
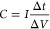
2in which *I* is the constant current and Δ*t* is the charge–discharge time corresponding to the operating voltage Δ*V*.

A GCD experiment measures electrode voltage as a function of time under a constant charging or discharging current and records the change law of the electrode’s potential with time during the entire charge–discharge process.^61^ For purely capacitive capacitors without redox reactions, the GCD curve resembles an almost symmetrical and linear curve (Figure 5e). The discharge process often resembles a curved line as can be observed in Figure 5f. This is often attributed to an increase in the active sites on the electrode surface.^62^

As a critical indicator to evaluate the performance of a supercapacitor, the stored energy directly reflects the capacitor’s ability to store charge, and it can be expressed as
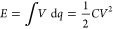
3where energy stored in the capacitor (*E*) is the work performed to charge the capacitor with the charge *q*. Supercapacitors have a high energy density, which will aid in the reduction of load and volume in energy storage systems.^48^ Consequently, power density, i.e., the rate of energy transfer, can intuitively reflect the rate of charge–discharge of supercapacitors, as given in eq 4:
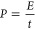
4where the amount of energy (*E*) that is delivered per unit of time (*t*) is correlated. Compared to batteries, the biggest advantages of supercapacitors are their ultrahigh power density and long lifetime. From these two formulas (eqs 3 and 4, it is evident that both energy density and power density are proportional to the operating voltage, which mainly depends on the choice of electrodes and electrolyte materials.^42^

EIS has been used extensively to quantify resistive components in the electrode and electrolyte by interpreting a “Nyquist plot”.^63^ A typical Nyquist plot can be divided into high-frequency and low-frequency regions. In the high-frequency region, the intercept of the *x*-axis represents the series resistance of the electrolyte, which is composed of electrode, electrolyte, and interfacial contact resistance.^64^ This is often followed by a semicircle arc in the high-frequency region representing the charge-transfer resistance as shown in Figure 5g. In the mid-frequency region, the presence of a 45° line indicates the presence of a Warburg impedance component induced by diffusion processes and penetration of electrolyte ions in the active substance, followed by a straight line close to 90° for supercapacitors and 45° for battery-type devices.^65^

### Ionic Conductivity of GPEs

The resistance of GPEs is often measured by sandwiching it between two metal sheets, and the ionic conductivity (σ) can be calculated using the following equation:
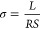
5where *L* is the thickness of the electrolyte layer, *R* is the bulk resistance and *S* is the cross-sectional area of the electrolyte. In fact, *R* represents the intersection of the line with the real axis of the Nyquist plot (*Z*_real_) (Figure 5h).

The performance of electrochemical energy devices, including their power and energy output, device voltage, and rate performance, is significantly influenced by the ionic conductivity of the electrolyte. Essentially, the water content, ion concentration, and nanopore structure of a polymer membrane all play crucial roles in the ion-conducting property of the electrolyte. Hydroxyl-rich biopolymers have an excellent ability to bind water, thereby enhancing salt dissolution. For example, negative carboxylic acid groups or sulfonated groups on the surface of cellulose derivatives have also been reported to promote counterion migration, boosting ionic conductivity (Figure 4c).^53^ Additionally, amphoteric polymers like betaine which have anionic and cationic counterions on the polymer chain might facilitate ion transport through triggering the dissociation of salts.

Additionally, small pore diameters typically lead to increased ion transport resistance, whereas large pore sizes might result in the passage of active material to and within the electrode and hence alter the overall performance of the device.^66^ In order to achieve the desired capacitive performance of supercapacitors, adequate pore size and homogeneous distribution of the pores are crucial to enable stable current transmission. Fortunately, biopolymer-based materials show unique advantages in terms of regulation of porous microstructure. For instance, tunable dynamic networks of cellulose nanofibrils (CNFs) are fabricated by swelling an anisotropically dewatered CNF gel in acidic salt solutions.^67^ A network is created by taking advantage of the extremely high aspect ratio of ultrathin CNFs. Modulation of the pore structure by using different concentrations of salt ions leads to a sharp increase in conductivity from 0.05 to 0.6 mS cm^–1^. Therefore, effective modulation of the pore structure of the polymer network is one of the key elements to obtain high ion transport. In addition, the phase separation within nanochannels formed by salt-induced polymer chain aggregation facilitates ion transport, as well as temperature and polymerization-induced phase separation that facilitates stable interfacial bonding to achieve stable ion transport.^68^

Macroscopically, all biopolymer materials are rendered electrochemically inert, with weak ionic conductivity. Precise grafting of functional groups and rational combinations with other highly conductive materials are the keys to realizing biopolymer-based gel electrolytes with high ion transport properties. The reactivity, electronegativity, ion adsorption, and dispersion of biopolymers prepared by different surface grafting approaches are different, thus requiring effective synergy with other components to achieve gel electrolytes with high performance. Some biopolymer materials can be combined with inorganic substances to protect biopolymer backbones, regulate hydrophilicity/hydrophobicity, and endow them with self-cleaning, antibacterial, and improved conductive properties. Lu et al.^69^ proposed the preparation of a high ionic conductivity (46.3 mS cm^–1^) cellulose hydrogel electrolyte by distributing bentonite nanoparticles uniformly on cellulose chains via stable coordination with −OH (Figure 4d). The lamellar structure of the bentonite nanosheets and the electronegativity of the cellulose/bentonite nanocomposites form an unobstructed ion channel to promote the migration of positive ions.

Table 1 summarizes the electrochemical performance of supercapacitors with a variety of biopolymer-based hydrogel electrolytes. The current performance parameters of biopolymer-based gel electrolytes, including energy density, power density, and so on, are illustrated in Figure 4e,f, which presents a comparison to conventional gel polymer electrolytes.

**Table 1:** Electrochemical Performance of Supercapacitors Incorporating Biopolymer-Based Hydrogel Electrolytes

Electrodes	Hydrogel electrolyte	Ionic conductivity	Specific/area capacitance	Energy density (*E*)	Power density (*P*)	Cyclability (capacity retention after cycling)	Ref
**EDLC**							
AC^a^//CC^b^	Sodium polyacrylate/CS^c^/KOH	80 mS/cm	35 F/g at 1 A/g	3.77 Wh/kg	225 W/kg	–	(31)
CC//CC	PANI/cellulose/PAM^d^	–	835 mF/cm^2^ at 1 mA/cm^2^	74.22 μWh/cm^2^	800 μW/cm^2^	96%@5000 cycling	(70)
Lignin/PAN/ECNFs	Lignin/KOH/PEGDGE	16.2 mS/cm	120 F/g at 1 A/g	4.49 Wh/kg	252 W/kg	99%@10,000 cycling	(71)
AC//AC	P(AA-*co*-AM)/gelatin	50 mS/cm	286.74 F/g at 0.2 A/g	39.09 Wh/kg	–	96%@10,000 cycling	(72)
AC//AC	CMC^e^/citric acid/CaCl_2_	64 mS/cm	309 F/g at 0.5 A/g	63.3 Wh/kg	–	86%@1000 cycling	(73)
AC//AC	PAM/CS/SiO_2_	51.3 mS/cm	140.1 F/g at 0.1 A/g	23.54 Wh/kg	110.1 W/kg	91%@10,000 cycling	(74)
AC//AC	P(AM-*co*-DMAEMA)/gelatin	13.6 mS/cm	163.6 mF/cm^2^ at 1 mA/cm^2^	–	–	86%@10,000 cycling	(75)
AC//AC	PAM/CS/H_3_PO_4_	100 mS/cm	106 F/g at 0.1A/g	–	–	97%@10,000 cycling	(76)
AC//AC	PVA/SA/PEG	65.4 mS/cm	103.6 mF/cm^2^ at 2 mA/cm^2^	14.39 μWh/cm^2^	1 mW/cm^2^	84%@6000 cycling	(77)
AC//AC	CS-PAM-Li_2_SO_4_	17 mS/cm	32F/g at 0.5A/g	8.7 Wh/kg	350.3 W/kg	–	(32)
AC//AC	PVA/agar-EMIMBF_4_-Li_2_SO_4_	43.6 mS/cm	28.8 F/g at 0.3 A/g	4 Wh/kg	150 W/kg	80%@10,000 cycling	(26)
AC//AC	CS	–	72 F/g at 1 A/g	32.6 Wh/kg	900 W/kg	98%@3000 cycling	(34)
CC//CC	Carrageenan/PAM- Li/K	87 mS/cm	93.0 F/g at 1 A/g	–	–	95%@20000 cycling	(78)
AC//AC	Lignin/gelatin	60 mS/cm	145 F/g at 0.5 A/g	4.67 Wh/kg	251.54 W/kg	81%@6000 cycling	(27)
AC//AC	Li- agar/PAM	45.7 mS/cm	84.7 F/g at 0.2 A/g	–	–	91%@1000 cycling	(79)
CC//CC	CMC/citric acid	–	441 F/g at 0.5 A g	72 Wh/kg	331 W/kg	91%@4000 cycling	(80)
CC//CC	Gelatin/CNF/Na_2_SO_4_	1.9 mS/cm	7.6 mF/cm^3^ at 0.02 mA/cm^2^	–	–	79%@2000 cycling	(81)
PAM/Ag-lignin/CNT	PAM/Ag-lignin/LiCl	84.71 mS/cm	148.8 F/g at 1 A/g	11.4 Wh/kg	4714.8 W/kg	89%@10,000 cycling	(82)
PPy-coated CNTs	PAM/protein/LiCl	16 mS/cm	246.8 F/g at 0.3 A/g	21.4 Wh/kg	2580 W/kg	80%@5000 cycling	(83)
Pure polypyrrole	Agar/PAM	–	183 mF/cm^2^ at 0.2 mA/cm^2^	–	–	95%@4000 cycling	(84)
PANI@CC	Lignin/NaOH	80 mS/cm	190 F/g at 0.25 A/g	9.35 Wh/kg	2157.3 W/kg	91%@10,000 cycling	(85)
PANI@CC	CS/SA/NaCl	51 mS/cm	234 F/g at 0.25 A/g	–	–	95%@1000 cycling	(86)
PANI nanowires	SA-borax/gelatin	30 mS/cm	185 F/g at 0.25 A/g	18.89 Wh/kg	100 W/kg	84%@10,000 cycling	(87)
Lignosulfonate/SWCNT	Cellulose/Li_2_SO_4_	–	292 F/g at 0.5 A/g	17.1 Wh/kg	324 W/kg	78%@3000 cycling	(88)
rGO gel	PAM/LiCl/WSCA	167 mS/cm	98.4 F/g at 3 A/g	8.7 Wh/kg	99.98 W/kg	91%@10,000 cycling	(33)
rGO paper	PVA/CMC	17.3 mS/cm	242 F/g at 0.25 A/g	87.9 Wh/kg	162.8 W/kg	84%@10,000 cycling	(89)
rGO/WO_3_	Gelatin/NaCl	250 mS/cm	70 mF/cm^2^ at 0.5 mA/cm^2^	30.28 mWh/cm^3^	7.67 W/cm^3^	96%@110000 cycling	(90)

**Pseudocapacitor**							
Zn//N/P-rGO	PAAK/CMC	35.2 mS/cm	190 F/g at 5 A/g	100.2 Wh/kg	487.5 W/kg	86.6% @8000 cycling	(91)
PANI/RGO/PMFT	PAM/BC	125 mS/cm	564 mF/cm^2^ at 1 mA/cm^2^	50.1 μWh/cm^2^	0.4 mW/cm^2^	92% @10,000 cycling	(92)
PEDOT:PSS/CNTs/PAM/SA	PAM/SA/Na_2_SO_4_	14.9 mS/cm	128 mF/cm^2^ at 1 mA/cm^2^	3.6 μWh/cm^2^	0.2 mW/cm^2^	79%@5000 cycling	(93)
N-rGO//N-rGO	Potassium polyacrylate/CMC	33.2 mS/cm	55 F/g at 0.3 A/g	311.4 W/kg	33 Wh/kg	88.2%@6000 cycling	(94)
N-rGO//N-rGO	DMSO/CS/LiTFSI	13.7 mS/cm	107.6 F/g at 1 A/g	62.9 Wh/kg	1025.5 W/kg	80%@5000 cycling	(95)

**Hybrid Supercapacitor**							
CoNiLDH@CC//AC@CC	PANa/BC/KOH	1.91 S/m	128 F/g at 2 A/g	25.6 Wh/kg	1200 W/kg	92%@10,000 cycling	(36)
AC//Zn	SA/PAA^f^/ZnSO_4_	–	255 mAh/g at 2 A/g	164 Wh/kg	1283 Wh/kg	95%@5000 cycling	(96)
AC//Zn	Borax/PVA/CNF	18 mS/cm	504.9 mF/cm^2^ at 0.5 mA/cm^2^	180 mWh/cm^2^	0.402 mW/cm^2^	95%@5000 cycling	(97)
N-rGO//cobalt carbonate hydroxide/N-rGO	Cellulose/NaOH	–	140 F/g at 1 A/g	45.3 Wh/kg	742 W/kg	93.9%@5000 cycling	(98)
MoO_3-*x*_ nanorods//AC	Na-alginate	–	42 mF/cm^2^ at 0.2 mA/cm^2^	21.20 μWh/cm^2^	0.18 mW/cm^2^	95%@10,000 cycling	(99)

a Activated carbon.

b Carbon cloth.

c Chitosan.

d Polyacrylamide.

e Carboxymethyl cellulose.

f Poly(acrylic acid).

### Mechanical Properties

Given the bending and compression associated with the utilization of supercapacitors, it is crucial for them to possess mechanical flexibility that enables them to endure diverse types of mechanical deformations without compromising their electrochemical performance.^100^ As an important aspect of supercapacitors, gel electrolytes can profoundly influence the mechanical properties of supercapacitors. Many biopolymers derived from natural polysaccharides rich in hydrophilic groups (e.g., −OH, −COOH) are advantageous for constructing stable polymer networks, largely due to their strong absorption affinity toward polar solvent molecules, as well as their ability to enhance mechanical properties.^12^

**Figure 6:**
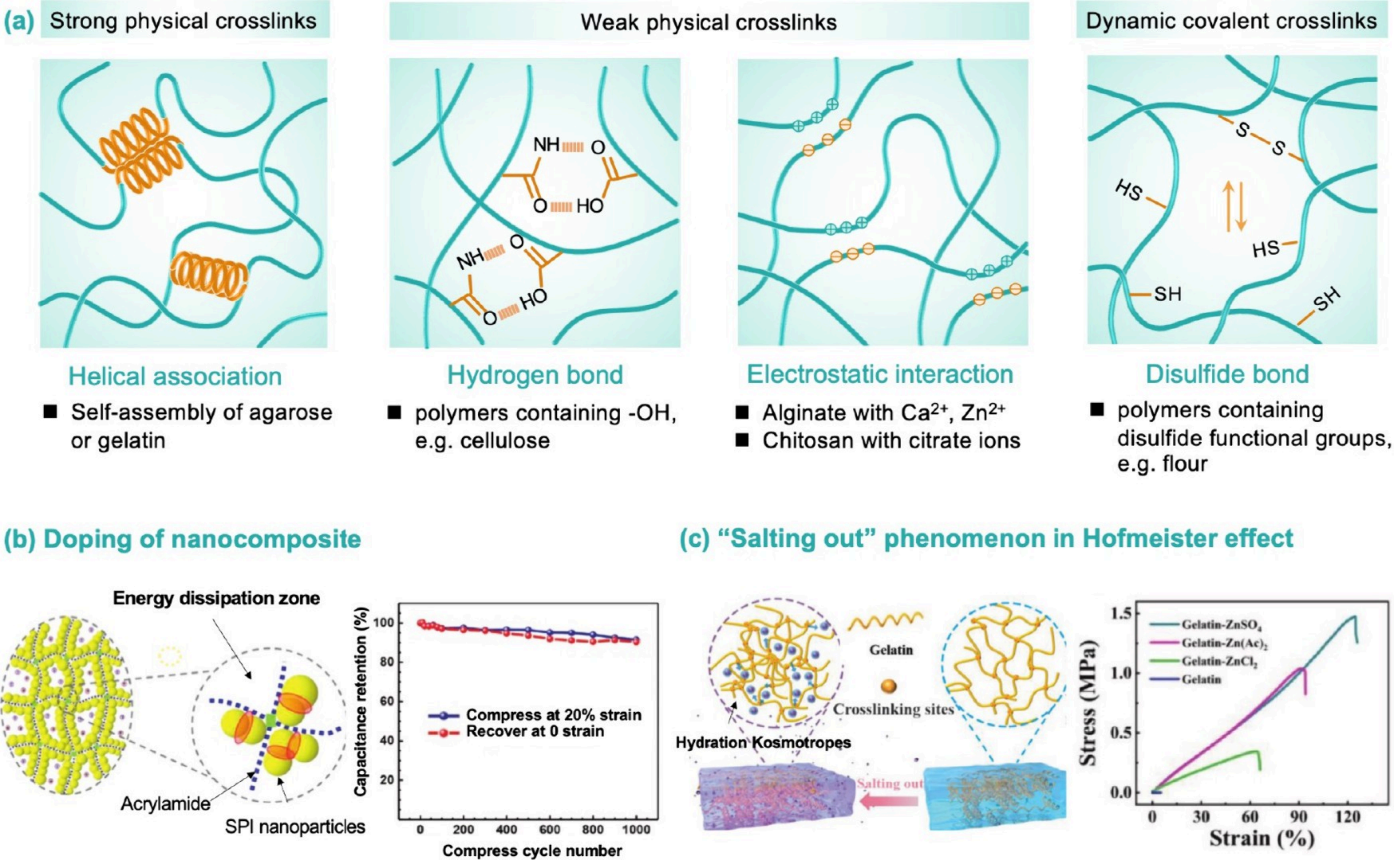
Schematic diagrams of biopolymer-based hydrogel electrolytes for supercapacitors with different energy dissipation mechanisms. (a) Cross-linking strategies of hydrogel for enhanced mechanical performance. (b) Doping of nanocomposite: interaction of soybean protein isolate (SPI) nanoparticles enhanced with a PAM hydrogel and its capacitance retention with compress–release cycles. Reused with permission under a Creative Commons CC-BY4.0 license from ref (83). Copyright 2023 Wiley-VCH. (c) Hofmeister effect: A gelatin-based hydrogel electrolyte fabricated through the “salting out” process in the Hofmeister effect, and the mechanical performance of different hydrogel electrolytes. Reused with permission from ref (101). Copyright 2023 Wiley-VCH.

The mechanical properties of gel electrolytes are largely dependent on cross-linking strategies (Figure 6a), which can be divided into permanent cross-linking (i.e., chemical bonding) and reversible cross-linking with physical interactions (e.g., hydrogen bonds, ionic bonds, hydrophobic associations, or polymer chain entanglements).^5^ As a representative type of tough hydrogels with high water content and superior mechanical strength, double network (DN) hydrogels have attracted great interest. These DN hydrogels have the ability to significantly improve both the strength and flexibility of biopolymer-based supercapacitors. Biopolymers can be readily introduced into the DN gel system and enable high mechanical properties while maintaining good electrochemical performances.

Among them, biopolymer materials can be used as a backbone to support the polymer network or as nanoadditives for energy dissipation nodes, both of which can optimize the mechanical properties of gels. Liu et al.^102^ reported a flexible, yet supertough, supercapacitor based on the highly effective energy dissipation of a DN hydrogel electrolyte, which was composed of covalently cross-linked polyacrylamide (PAM) and Al^3+^ ionically cross-linked alginate. The resulting supercapacitor could endure multiple twisting, hammering, and cutting processes up to 1000 times without significantly impacting its capacitance. Similarly, an agar/hydrophobically associated PAM DN hydrogel was fabricated for an all-polymer supercapacitor,^84^ in which the fragile network in the DN hydrogel acts as a sacrificial bond for destruction and dissipation of energy, making the entire material tough.^79^

In addition to the DN design, high-density dynamic ionic interactions are also considered an appropriate approach to fabricate hydrogel electrolytes with excellent strength. For instance, Yuan et al.^103^ introduced a chitosan derivative-based flexible hydrogel, which was synthesized by a one-step copolymerization of negatively charged monomers (acrylic acid) in a positively charged natural polysaccharide matrix under the partial shielding effect of NaCl solution. The resulting freeze-dried hydrogel assumed a compact, granular structure via strong interchain ionic bonds. Besides enhanced tensile properties (large strain of 920%, Young’s modulus of 2.53 kPa), ionic bonds and interchain entanglements also enable the supercapacitors to endure very high voltage/current change rates.

Recently, dissolved cellulose as a green reinforcing filler together with lignin and citric acid were added into a PAA network for zinc-ion engineered plant-based multifunctional hydrogels by Lyu et al.^104^ Plant-based functional ingredients (cellulose, lignin, and citric acid) ensure strong adhesion, antibacterial activity, and good biocompatibility of the biopolymer hydrogels, forming a porous structure. The long-chain cellulose macromolecules dissolved in ZnCl_2_ solution can form a cross-linking network via covalent and non-covalent bonds with PAA, endowing the hydrogel with outstanding mechanical properties (800 kPa at 520%). The assembled supercapacitor demonstrated a long-duration cycling lifespan (10,000 cycles) with a capacity retention of 85.6%.

Moreover, nanomaterials can also be introduced into hydrogels to enhance the mechanical performance. A nanocomposite hydrogel was fabricated for a reversibly compressible quasi-solid-state supercapacitor.^83^ Soybean protein isolate (SPI) nanoparticles were introduced into a PAM network to form a cross-linking structure, which could effectively disperse applied stress and dissipate energy (Figure 6b). The pore image of PAM/SPI hydrogels indicated that the SPI ratio mainly dictated the thickness of pore walls, effectively enhancing the mechanical strength of polymer chains. As expected, the obtained quasi-solid-state supercapacitor device could undergo multiple compressive cycles and maintain high capacitance retention for 1000 compression cycles even at strain levels as high as 80%.

The above introduction of dynamic bonds or composites with synthetic polymers to jointly construct a DN is an effective method to enhance the mechanical properties of natural polymer hydrogels. In particular, in the absence of synthetic polymers, Wang et al.^105^ proposed the strategy of integrating saline solution (sodium sulfate) and micro-nano enhancement to successfully construct gelatin-based hydrogels with ultrahigh strength. The saline immersion process based on the Hofmeister effect can induce the aggregation of protein molecules in gelatin and the generation of hydrophobic cross-links, thereby increasing the strength of hydrogels. The strength and elongation at the break of the hydrogel reached 0.73 MPa and 250%, respectively. The Hofmeister effect is generally interpreted as the solubility of synthetic and natural polymers in aqueous systems, which can be affected by the type of salt ions.^106^ On the path to pursuing high-performance supercapacitors, there are still challenges in optimizing the mechanical properties and electrochemical performances of gel electrolytes. Based on the “salting out” phenomenon in the Hofmeister effect, a kind of gelatin-based hydrogel electrolyte was developed by soaking a gelatin hydrogel in a ZnSO_4_ salt solution (Figure 6c).^101^ Inorganic salts induce the hydrophobic effect of polymer segments, making polymer segments denser and providing good water retention, while strengthening hydrogen bonds between polymer segments. As a result, the gelatin–ZnSO_4_ electrolyte demonstrates a high breaking strength of 1.5 MPa and stable electrochemical performance in an assembled supercapacitor, which could sustain 7500 charge–discharge cycles. In addition to the Hofmeister effect, there are electrostatic interactions as well as metal ion coordination interactions associated with salt ions that also can modulate the mechanical and electrochemical properties of the gels.^34^ The basic properties of ions (radius, charge, structure) as well as the concentration have a great influence on the overall performances of ion-conducting hydrogels forming supercapacitors.^107^ Therefore, the reasonable selection of salt ions is the key to obtaining high-performance supercapacitors with excellent mechanical properties.

### Self-Healing Ability

Self-healing refers to the ability of a material to regenerate mechanical, structural, and functional properties without external intervention after a deformation event.^108^ Ideally, a self-healing material can withstand several cycles of deformation and regeneration. Although gel electrolytes with excellent comprehensive mechanical properties have been developed, the damaged chemical networks involved in the main energy dissipation of these hydrogels often cannot heal or recover once they have suffered structural damage under high strain. This results in deterioration of electrochemical performances and permanent loss of the mechanical properties of the gel electrolytes. To overcome these limitations, an effective approach is to introduce reversible physical or chemical cross-links, including hydrogen bonds,^26^ hydrophobic interactions,^97^ ionic bonds,^109^ and electrostatic interactions,^103^ to replace chemical bonds with sacrificial bonds. Supercapacitors based on self-healing hydrogel electrolytes can repair their physicochemical structure and electrochemical properties through dynamic and reversible cross-linking when subjected to mechanical damage, significantly extending their service life.^110^

**Figure 7:**
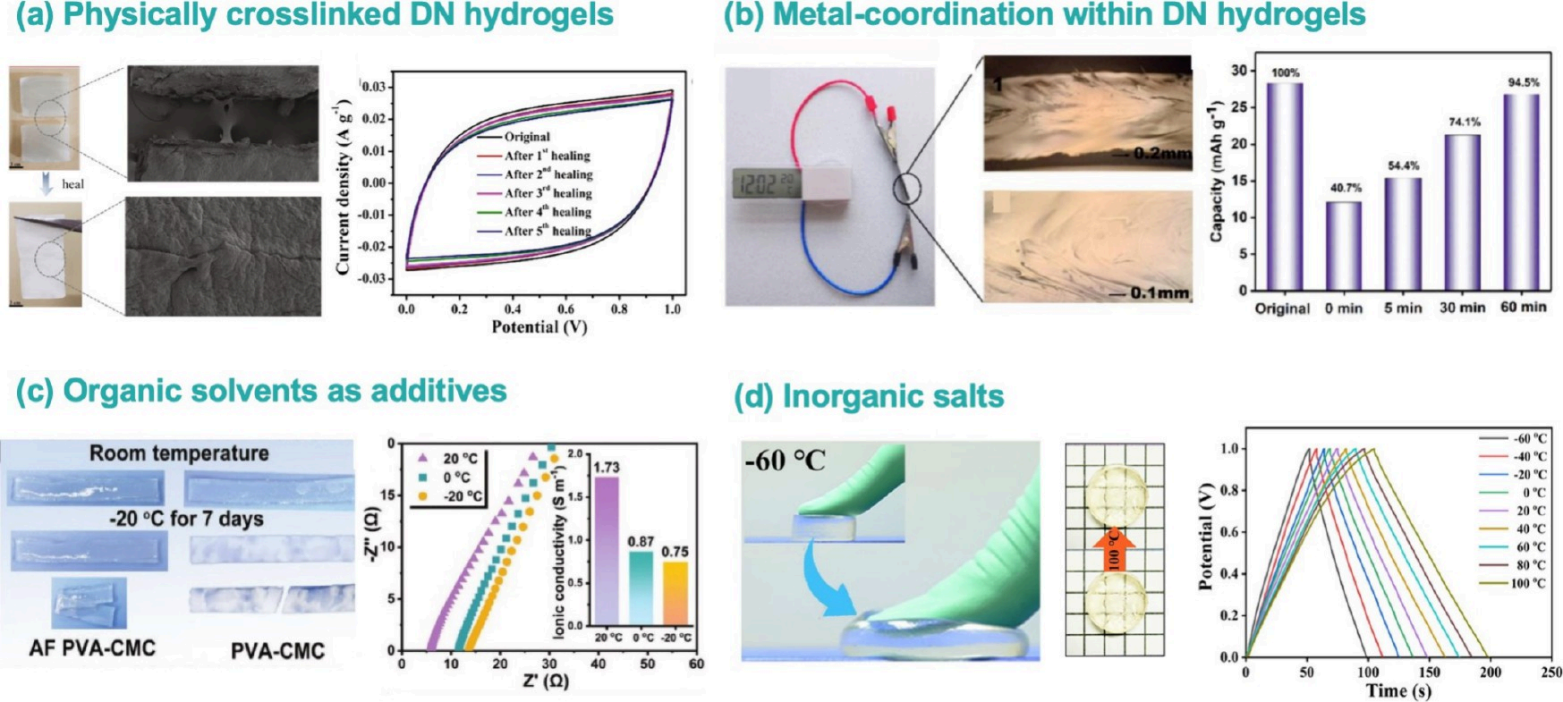
General strategies for biopolymer-based gel electrolytes to improve the self-healing and antifreezing ability. (a) Physically cross-linked DN hydrogels: optical images and SEM images of the self-healing process for the optimized DN hydrogel electrolyte, and the corresponding cyclic voltammograms for the obtained supercapacitor. Reused with permission from ref (26). Copyright 2021 Elsevier. (b) Metal-coordination within DN hydrogels: optical microscope images and capacity recovery efficiency after healing for different times. Reused with permission from ref (109). Copyright 2021 Elsevier. (c) Organic solvents as additives: schematic diagrams of the hydrogel resistance at low temperatures, and their ionic conductivities at different temperatures. Reused with permission from ref (89). Copyright 2022 Wiley-VCH. (d) Inorganic salts: schematic diagrams of the hydrogel resistance at −60 and 100 °C, and GCD curves at different temperatures. Reused with permission from ref (76). Copyright 2021 Elsevier.

As previously discussed, self-healing DN hydrogels are typically based on hydrogen-bond interactions and/or chain entanglement. They can be composed of hydrophilic cross-linked polymer networks and biopolymers (e.g., nanocellulose and agar) and exhibit higher mechanical properties and excellent self-healing properties compared to traditional single-network hydrogels.^5^ For instance, a zinc-salt-containing borax-cross-linked PVA/nanocellulose hydrogel electrolyte with high strength was produced by Chen et al.^97^ through the synergism of borax-mediated multicomplexation. The borax in hydrogel electrolytes enables dynamic association between PVA and nanocellulose. When the two parts of the supercapacitor were put together and regenerated at room temperature, much of the capacity could be recovered in 60 min. Similarly, Peng et al.^26^ introduced agar into the PVA polymer network by a simple one-pot physical cross-linking and freezing/thawing method to prepare a DN gel electrolyte (Figure 7a). Benefiting from the hydrogen-bond-associated chain entanglement of agar spiral bundles as well as a second network established by crystallites formed with PVA hydrogel, the gel electrolyte can recombine spontaneously after a few minutes without a noticeable boundary around the healing area. Accordingly, the supercapacitor assembled with this DN hydrogel electrolyte still showed rectangular CV curves after five cutting/healing cycles. It is worth mentioning that the dynamically cross-linked natural polymer-based gel electrolytes exhibit more rapid self-healing properties. Peng et al.^87^ constructed a SA-borax/gelatin DN conductive hydrogel composed by dynamic cross-linking between SA and borax via borate bonds, as well as hydrogen bonding between SA and amino acids in gelatin, which endows the hydrogel with rapid self-healing performance (restoring to the original state in 20 min with only slight capacitance loss).

Additionally, dynamic ionic interactions are regarded as a good strategy for achieving rapid self-healing. Zhang et al.^109^ introduced anhydrous betaine and zinc sulfate heptahydrate into the PAA system to prepare zwitterionic hydrogel electrolytes for zinc-ion hybrid supercapacitors (Figure 7b). The carboxyl and zinc ions bound to the PAA chains created reversible, dynamic ionic interactions. As a result, the healing efficiency reached 83.69% after healing for 20 min.

### Temperature Tolerance

The obvious drawback of many conventional hydrogel electrolytes is their instability. Hydrogel electrolytes have been associated with acute temperature sensitivity (e.g., freezing of gel and loss of ionic conductivity in extreme cold and/or sweltering climates) which often results in reduced performances (e.g., severe loss of capacity) and severely hinders the practicality of hydrogel electrolytes for supercapacitors.^111^ In general, hydrogel electrolytes employ free water as an ionic conduction medium for superior ion transport.^112^ However, the free water has a tendency to freeze at subzero temperatures and evaporate at high temperatures, which consequently restricts the electrical conductivity.^113^ The freezing of water molecules in polymer matrices will result in significantly lower water motility and precipitation of electrolytic salts, limiting the polymer’s ability to self-heal and the device’s electrochemical performance at low temperatures.^3^ Thus, significant efforts have been applied to the challenge of broadening the working temperature range of gel electrolytes.

To date, one effective technique for broadening the adaptive temperature range of hydrogels is to introduce an additive (e.g., an organic solvent or metal salt) into the aqueous medium of the polymer networks. For organic solvents, the key is to break the hydrogen bonds between water molecules and to form molecular clusters composed of solvents and water.^114^ For example, a freeze-tolerant hydrogel electrolyte was developed by soaking the semi-interpenetrating PVA–carboxymethyl cellulose network in an aqueous solution of ethylene glycol containing the Zn^2+^ ion.^89^ The incorporation of ethylene glycol as a cryoprotectant in the polymer matrix effectively inhibited the growth of ice crystals. The resulting device achieved a high degree of flexibility and good energy densities (87.9 Wh/kg at 20 °C and 60.7 Wh/kg at −20 °C) even at temperatures as low as −20 °C (Figure 7c), although, admittedly, the presence of organic solvents significantly weakened the adhesion and toughness of hydrogel electrolytes and hindered the migration of ions.^115^

For low-temperature resistances, attempts have been made to introduce inorganic salts into hydrogel systems to prepare antifreeze hydrogels with high ionic conductivity for supercapacitors.^116^ Xu et al.^76^ utilized phosphoric acid and water as a mixed solvent to dissolve chitosan, which was introduced into the chemically cross-linked network of PAM to prepare a hydrogel electrolyte with high thermostability (Figure 7d). Among them, phosphoric acid molecules combined with water form multiple hydrogen bound complexes, thereby inhibiting the crystallization of water. The supercapacitors exhibited superior charge–discharge stability in a wide temperature range from −60 to 100 °C. Other salt ions can also immobilize water molecules to achieve high thermostability. A carrageenan/PAM DN hydrogel with a mixture of LiCl and KCl solutions was fabricated^78^ which achieved a high conductivity of 1.9 S/m at −40 °C and capacitance retention of 95.6% after 20,000 cycles at −40 °C. It has been demonstrated that high concentrations of LiCl can confer good ionic conductivity, antifreeze performance, and better water retention capacity to non-ionic PAM hydrogels. However, a high salt concentration can coagulate ionic polysaccharides and make it difficult for DN hydrogels to polymerize. A recent study has shown that dynamically cross-linked alginate networks grafted with dopamine could tolerate a high concentration of KCl.^117^ The high salt tolerance is related to the abundant carboxyl groups in the alginate, which allows the chains of the biopolymer to associate with cations, alleviating the “salting out” effect. At −10 °C, the electrolytes maintained an ionic conductivity of 85.7 mS cm^–1^ at KCl concentrations as high as 3 mol L^–1^.

Another effective method to increase the temperature adaptation range of a hydrogel is to substitute water with thermally stable ionic liquids.^118^ The resulting ionogels, which have high ionic conductivity, a broad electrochemical potential window, and strong thermal stability, are promising candidate electrolytes for flexible supercapacitors. For instance, a cellulose hydrogel with an organic solution of superbase/DMSO/CO_2_ that exhibited excellent thermostability at temperatures as high as 100 °C was developed based on the reversible chemistry of CO_2_ with alcohols.^119^ After 50 cycles of charging and discharging at different temperatures (5–100 °C), the capacity was almost unchanged when the temperature was restored to 25 °C.

## Current Challenges in Biopolymer-Based Hydrogel Electrolytes

### Recyclability and Sustainability of Supercapacitors

So far, a considerable number of studies have focused on the performance aspect of the prepared supercapacitors including their mechanical properties, temperature tolerance, and durability. In contrast, few investigations have been conducted on the disposal of spent supercapacitors. Currently, the majority of the electrolytes and electrodes are non-recyclable and often not readily degradable, adding to the burden on the deteriorating global environment. In comparison to other materials, gel electrolytes which utilize biopolymer as the building blocks are comparatively easy to recover and degrade. Of course, this also requires a reasonable selection of materials to ensure its stable electrochemical performances after recycling.

**Figure 8:**
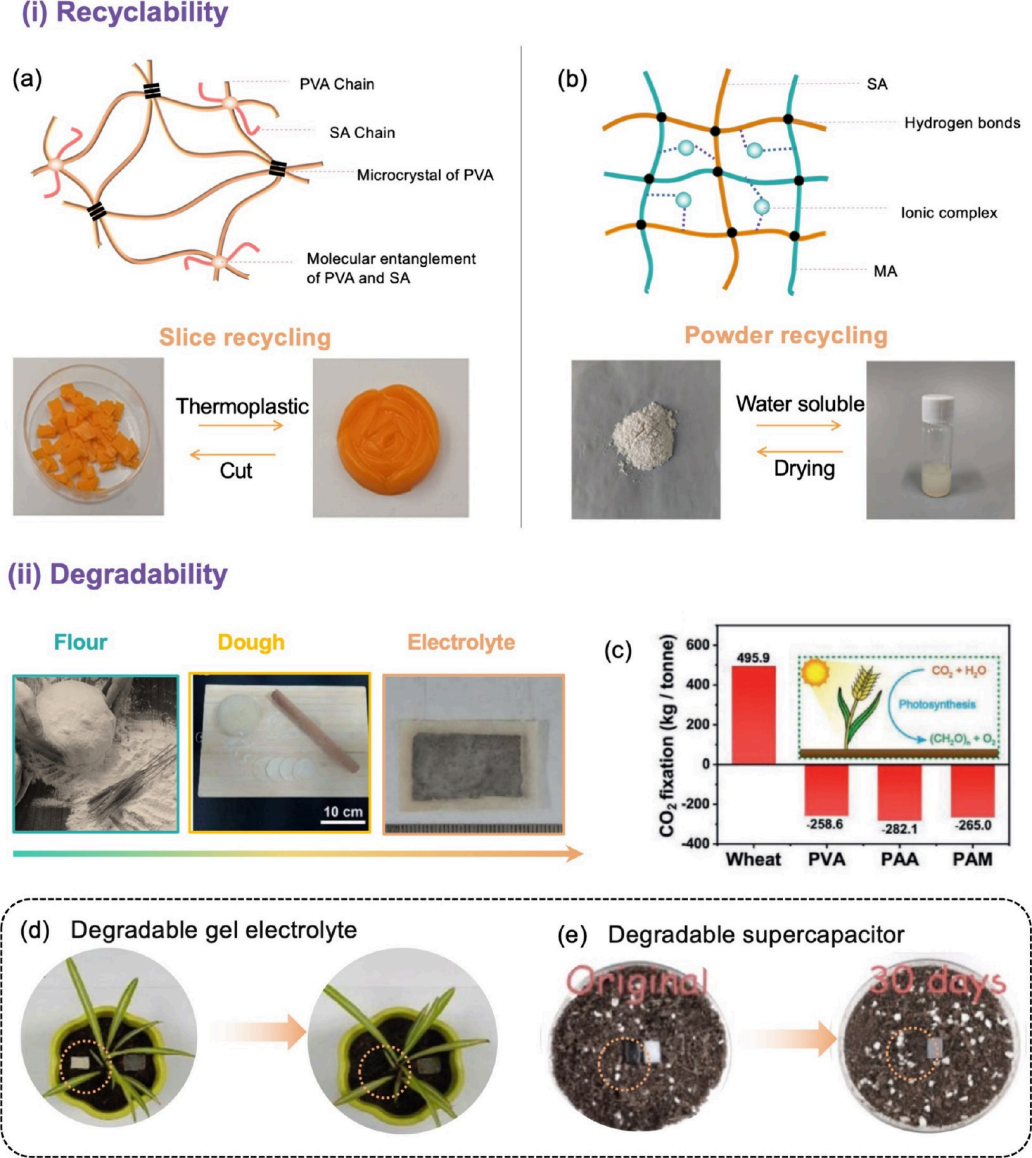
Current performance optimization strategies for biopolymer-based gel electrolytes. (a) An internal structure diagram of PVA/SA/PEG organogel electrolytes and photographs showing the thermoplasticity. Reused with permission from ref (77). Copyright 2021 American Chemical Society. (b) Structure and photographs of the regeneration of hydrogels. Reused with permission from ref (96). Copyright 2021 Wiley-VCH. (c) Comparison of CO_2_ fixation between wheat, PVA, PAA, and PAM. (d) Demonstration of the degradability of flour-based gel electrolytes. Reused with permission from ref (122). Copyright 2019 Wiley-VCH. (e) Demonstration of the degradability of all-flour-based supercapacitors. Reused with permission from ref (121). Copyright 2021 Wiley-VCH.

This challenge can be addressed by developing recyclable gels that utilize dynamic and reversible cross-linked structures. An efficient way to generate high-strength, recyclable hydrogels is to employ synthetic polymers with high thermal stability, such as PVA, as a matrix and natural polymers to regulate the network topology. Hu et al.^77^ proposed that dehydration could be used to enable effective recycling of poly(vinyl alcohol)/sodium alginate/polyethylene glycol (PVA/SA/PEG) organic hydrogel electrolytes. The materials could then be rehydrated and reused by soaking in a saturated NaCl aqueous solution (Figure 8a). The SA and PVA interact to generate a semi-interpenetrating polymer network wherein a large number of hydrogen bonds can be created within the polymer chains as a result of PEG’s bridging effect. The solution can be regenerated after chopping, heating, and remelting the organic hydrogel, which still can exhibit good mechanical and electrical conductivity even after 20 heating–remolding cycles. Similarly, a physically cross-linked starch/PVA/glycerol/CaCl_2_ organogel, assembled with commercially available activated carbon as the electrodes for a flexible all-solid-state supercapacitor, was investigated by Lu et al.^120^ Due to the dynamic breakage and recovery of non-covalent bonds, an important and intriguing characteristic of the organogel is its favorable thermoplasticity. This property enables the organogel to be easily transformed into various shapes at 116 °C and endows the organogels with the ability to be recycled. In addition to heating the hydrogel to reshape it for recycling, some researchers have proposed powdering the gel and molding it in a water dispersion to enable recyclability. A recyclable ionically conductive hydrogel (ZnSO_4_/SA/PAA) was developed for a flexible hybrid supercapacitor (Figure 8b).^96^ Due to the dynamic reversible interactions constructed by hydrogen bonding and ionic complexation of the electrolyte, it could easily regenerate the hydrogel electrolyte after powdering (fast recovery in 3 min) and maintain stable electrochemical performance (capacity retention of 95.3% after 5000 charge–discharge cycles).

In light of their superior ionic conductivity and mechanical qualities, polymer gel electrolytes are widely employed in an array of energy storage devices. However, they are commonly non-biodegradable and will lose flexibility and electrochemical performance over the dehydration/rehydration process.^73^ Since the electrolytes and/or electrode materials widely used in flexible supercapacitors are corrosive or difficult to degrade, a new type of renewable, environmentally friendly, low-cost biomass-based flexible solid-state supercapacitor was fabricated based on degradable lignin/SWCNT hydrogel electrodes and a polymer gel (cellulose/Li_2_SO_4_) electrolyte.^88^ The assembled supercapacitor showed excellent electrochemical stability with 80.1% retention of the initial capacitance after 10,000 cycles. Furthermore, a fully cellulose hydrogel electrolyte was prepared by integrating a large number of H^+^ into a cellulose matrix and then immersing it in a salt solution to generate robust ionic cross-linking networks.^73^ This all-cellulose-based electrolyte showed a good ionic conductivity of 62 mS cm^–1^, good recyclability, and full degradation within 8 days. Yun et al.^90^ achieved highly conductive hydrogels by extracting gelatin from pig skin and soaking it in LiCl solution. They exhibited a high conductivity of 250 mS cm^–1^, and the specific capacitance of the resulting supercapacitors assembled with carbonized mulberry paper reached up to 450 mF cm^–2^. More interestingly, edible flour which mainly comes from wheat can also be used as a raw material to fabricate electrolytes for supercapacitors.^121^ As shown in Figure 8c, one ton of wheat plants can fix 495.9 kg of CO_2_ during their growth while the production of synthetic polymers results in the emission of a significant amount of CO_2_. A sustainable dough-based gel electrolyte with high biosafety and environmental friendliness was developed by Wang et al.^122^ The dough electrolyte material exhibited a stable structure at high temperatures and in a dehydrated state, because three-dimensional networks of gluten protein were connected by abundant disulfide bonds. After completely dehydrating and subsequently resoaking in a saline solution for 10 min, more than 98% of the original capacitance could be restored. The dough electrolyte could be gradually degraded by microorganisms within 16 days, showing both excellent recyclability and biodegradability (Figure 8d,e). Moreover, recent research has also explored the use of soy sauce in preparing edible gel electrolytes for supercapacitors,^123^ offering an additional inspiration for sustainable development through the combination of food and energy.

In most cases, polymer matrices swollen in acidic (e.g., H_3_PO_4_, H_2_SO_4_), alkaline (e.g., KOH), or neutral (e.g., LiCl, Na_2_SO_4_) aqueous media can be assembled as gel electrolytes for flexible supercapacitors. However, although biopolymers are employed to create environmentally friendly gel electrolytes, the inevitable usage of toxic and/or environmentally hazardous salt ions as a conducting medium greatly decreases the biocompatibility of biopolymer-based supercapacitors. Without introducing additional salt, Cevik et al.^80^ demonstrated the preparation of high ion-conducting biopolymer hydrogels through intercalating *Hibiscus sabdariffa* into a sodium carboxymethyl cellulose/citric acid system. In this system, both carboxymethyl cellulose and *H. sabdariffa* can provide ion migration that occurs via the H_3_O^+^ and Na^+^ ions from the mixture. Zhao et al.^86^ reported a method in which sodium chloride was added to two biopolymer solutions (one being chitosan, the other being SA) with opposite charges. Upon mixing the solutions, a semidissolved and acidified sol–gel was created. Strong electrostatic interactions formed between the two biopolymers, generating rapid ion migration channels. It endowed the solid-state supercapacitor with an excellent specific capacitance of 234.6 F g^–1^ at a scan rate of 5 mV s^–1^ and a capacitance retention of 95.3% after 1000 GCD cycles. Despite all these advances in the environmental friendliness and degradability of biomass-based hydrogels, it remains essential to pay attention to their capacitance loss over time and their environmental stability with respect to temperature and moisture effects.

### Stable Electrolyte–Electrode Interface

Since all electrochemical redox reactions and most electron/ion transport occur at the interface of the electrode and electrolyte, a homogeneous and stable interface structure with high electrochemical activity is essential. In gel electrolyte systems, the main strategies, including the incorporation of additives,^124^ heteroatom doping,^125^ and the construction of heterogeneous interfaces,^126^ have been well developed to enhance the interfacial capacitance.

**Figure 9:**
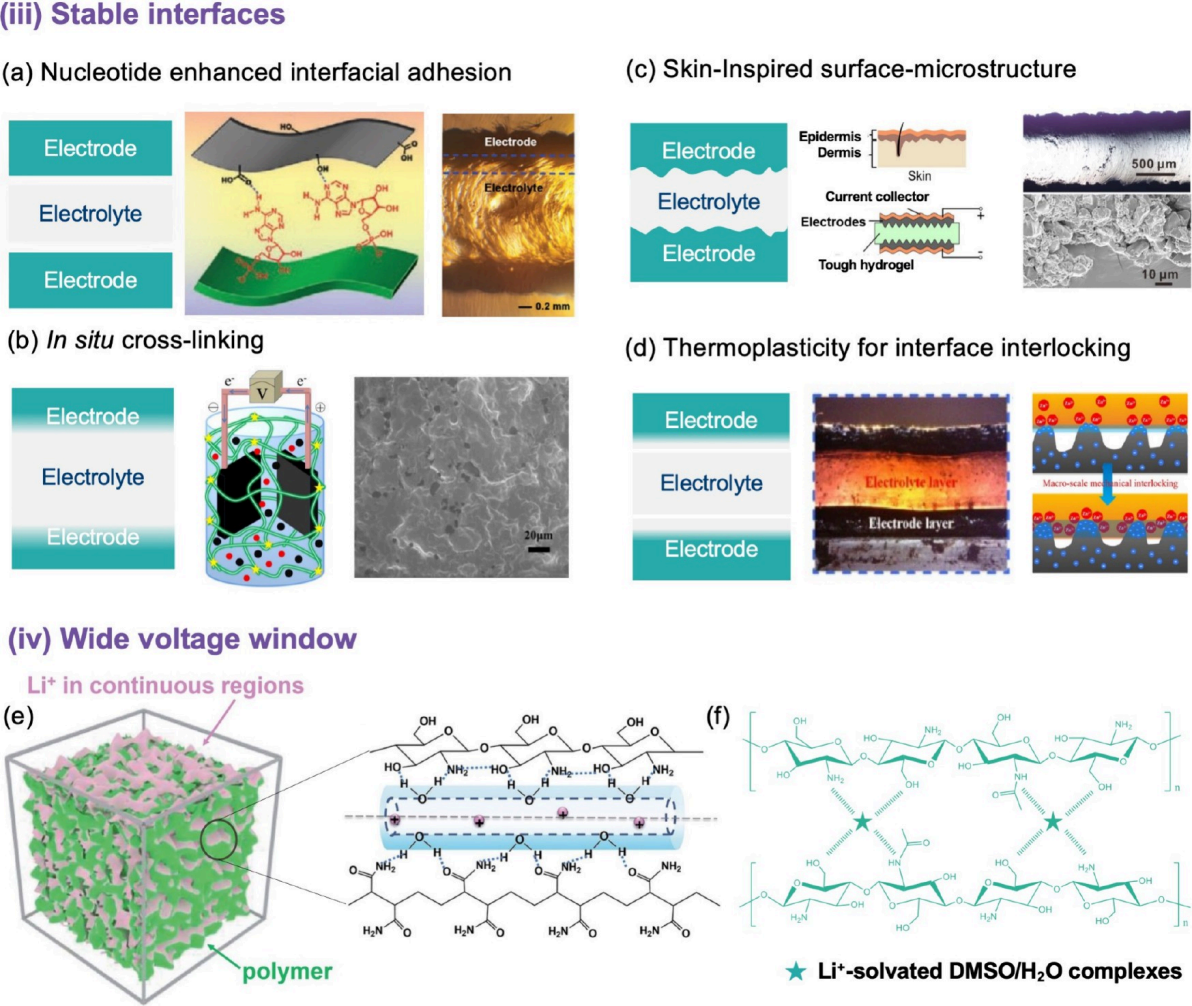
(a) Adenosine monophosphate-tackified P(AM-*co*-DMAEMA)/ gelatin double-network organogel electrolyte with strong adhesion. Reused with permission from ref (75). Copyright 2021 Wiley-VCH. (b) All-solid-state hydrogel supercapacitors fabricated through an *in situ* low temperature cross-linking method. Reused with permission from ref (127). Copyright 2019 The Royal Society of Chemistry. (c) Skin-inspired surface-microstructured tough hydrogel electrolytes for stretchable supercapacitors. Reused with permission from ref (128). Copyright 2019 American Chemical Society. (d) Thermoplastic-recycled gelatin/oxidized starch/glycerol/ZnCl_2_ organogels. Reused with permission from ref (129). Copyright 2023 American Chemical Society. (e) Demonstration of the synthesis and structural architectures of the water-in-salt electrolytes. Reused with permission from ref (74). Copyright 2019 The Royal Society of Chemistry. (f) Schematic illustration of the inner cross-linking method for the “dimethyl sulfoxide/water-in-salt”-based chitosan gel electrolyte. Reused with permission from ref (95). Copyright 2021 Wiley-VCH.

In an attempt to rectify issues of interfacial contact deformation resistance between the electrolyte-electrode interface, an all-in-one supercapacitor was prepared by Zhang et al.^75^ The supercapacitor was prepared by the direct assembly of a nucleotide-tackified poly(acrylamide-*co*-2-ethyl methacrylate)/gelatin DN organo-hydrogel electrolyte, which was connected to an activated carbon/carbon cloth electrode. The incorporation of adenosine monophosphate and the physically cross-linked gelatin network significantly enhanced the adhesion and mechanical performances of the organo-hydrogels (Figure 9a). The integrated supercapacitors displayed an areal specific capacitance of 163.6 mF cm^–2^ and a low interfacial contact resistance of 0.56 Ω. Likewise, carbon nanotube dispersions and LiCl were introduced into a hydrogel matrix to prepare all-hydrogel supercapacitors,^82^ in which Ag@lignin nanoparticles are homogeneously integrated in a polymer matrix that acts as a binder. Based on the non-covalent interaction of the rich hydroxyl and catechol groups of Ag@lignin nanoparticles, the supercapacitor exhibits tough interfacial adhesion (28 kPa) and excellent cycle stability (capacitance retention rate of 89.9% after 10,000 charge–discharge cycles). Moreover, Li et al.^92^ fabricated an all-solid-state supercapacitor by employing bacterial cellulose nanofiber-reinforced PAM as the hydrogel electrolyte and a graphene-encapsulated polyester fiber loaded with polyaniline as the flexible electrode. Thus, a stand-alone 3D porous composite with bacterial cellulose as a reinforced scaffold, interacting with the PAM chain through hydrogen bonding and physical cross/interleaved effects, was prepared. This architecture can promote the diffusion of electrolyte ions and alleviate the mechanical stress caused by deformation.

Recently, there have also been reports in which a stable electrode–electrolyte interface is achieved by *in situ* cross-linking. For example, Yan et al.^127^ employed *in situ* low-temperature cross-linking to combine novel cellulose-based hydrogel electrolytes with a N-doped graphene hydrogel electrode for all-solid-state asymmetric supercapacitors (Figure 9b). As expected, it delivered a high energy density of 45.3 Wh kg^–1^ at a power density of 742.0 W kg^–1^. The hydrogen bonding and epichlorohydrin cross-linking in cellulose/NaOH hydrogels ensured their high specific capacitance and energy density. The preparation of this material via *in situ* cross-linking prevents the formation of bubbles in the hydrogel by anchoring the electrolyte directly to the electrode. Similarly, an integrated electrode–electrolyte structure was constructed by *in situ* cross-linking with no extra binder to prepare a “dimethyl sulfoxide/water-in-salt”-based chitosan hydrogel electrolyte,^95^ which resulted in a maximum energy density of 62 Wh kg^–1^ at a power density of 1025 Wh kg^–1^. However, the *in situ* cross-linking technique frequently results in the inevitable swelling of hydrogel electrolytes and ion leakage, weakening the mechanical characteristics and conductivity of the resulting hydrogel electrolytes.

Macroscopic mechanical interlocking is an effective way to improve the strength of the interface between a polymer matrix and porous materials. In order to overcome delamination caused by a mismatch of the electrode–electrolyte interface during stretching/compressing, a tough agar/PAM/LiCl hydrogel with a dentate microstructured surface was reported, which was rubbed mechanically to improve interface adhesion, realizing the fabrication of a stretchable supercapacitor (Figure 9c).^128^ The charge-transfer resistance (which is related to the interfacial resistance) declined from 3.4 to 1.4 Ω. This indicates that the incorporation of surface-microstructured hydrogel electrolytes could result in durable supercapacitor materials. In addition, Liang et al.^129^ achieved thermoplastic characteristics by constructing gelatin/starch oxide/glycerol/ZnCl_2_ organic hydrogels and assembling them with activated carbon electrodes to form supercapacitors, which allow the establishment of gel–sol transition-induced interlocking at the interface (Figure 9d). Because of the thermally induced phase transition of gelatin, these organic hydrogel fragments were softened and penetrated into the interstices of porous materials at 65 °C, achieving good interfacial compatibility.

### Wide Voltage Window

A sustainable economy necessitates trade-offs between economic, environmental, and social values in order to maximize resource utility. The development of sustainable societies is heavily constrained by the contradiction between what is environmentally beneficial and what is of high performance and thus economically beneficial. For instance, many eco-friendly energy storage materials possess narrow electrochemical windows and extremely high concentrations of expensive aqueous electrolytes.^130^ Given the immediate need for sustainable and cost-effective energy storage, the improvement of energy densities of aqueous supercapacitors is highly desirable. Due to their wide electrochemical stability window (ESW), concentrated aqueous electrolytes, also known as water-in-salt electrolytes (WISEs), have been particularly popular.^131^ As a high-concentration water-based electrolyte, a WISE effectively alleviates the thermodynamic limitation of water and shows a wide ESW (∼2–3 V), far exceeding that of traditional water-based electrolytes. Meanwhile, maintaining the inherent advantages of traditional water-based electrolytes, including their safety, low cost, environmental friendliness, and satisfactory ionic conductivity, further improves the energy density and rate capability of the supercapacitors.^132^

High concentrations of lithium bis(triiodomethanesulfonyl)imide (LiTFSI) in WISEs effectively convert any available water molecules into solvent-sheath structures with dissolved cations, leaving little to no free water remaining, leading to an expanded ESW approaching the thermodynamic limit of water electrolysis.^133^

Usually, the polymer acts as a framework in the electrolyte and cannot accommodate ultrahigh ionic concentrations, restricting its electrochemical performance. However, polymer loadings can be reduced by incorporating biomass materials with abundant active sites, to achieve high-salt-loaded aqueous electrolytes. A novel PAM–chitosan-based gel electrolyte was designed by direct copolymerization with acrylamide, chitosan, and tetraethoxysilane (Figure 9e).^74^ To achieve ultrahigh salt loadings in hydrogels, the hydrogel polymer network with abundant hydroxyl and amino groups was combined with water molecules in a WISE, forming a linear framework that facilitates ion movement. The PAM–chitosan-based WISE resulted in high ionic conductivity (51.3 mS cm^–1^), with an operating voltage of 2.6 V. Thus, the WISE electrolyte strategy is indeed effective in improving the energy density of energy storage devices. Wang et al.^95^ successfully prepared hydrogel electrolytes using dimethyl sulfoxide as the solvent by supramolecular complexation between the Li^+^-solvated complex and the chitosan chain (Figure 9f). The 3D continuous chitosan network with well-distributed bound water allows cationic Li^+^ and anionic TFSI^–^ to easily separate without having to overcome the strong Coulombic attractions, which contributes to the enhanced ionic conductivity.

A novel hybrid “water in salt” solution of Zn(CH_3_COO)_2_ and CH_3_COOK was introduced into the potassium polyacrylate/sodium carboxymethyl cellulose hydrogel matrix for the construction of aqueous zinc-ion hybrid supercapacitors.^91^ Many of the acetate anions were directly coordinated with K^+^ and Zn^2+^, resulting in ion aggregation and transition from “salt in water” to “water in salt”, so that the activity of free water is effectively inhibited and alleviates the formation of dendrites, achieving a wider voltage window. Many studies have been devoted to improving the conductivity of WISE such as reducing the concentration of WISE, preparing mixtures with organic solutions, or optimizing the salts used.^134^ The problem of reduced conductivity at low temperatures can be alleviated by using a mixture of organic solvents. Therefore, the introduction of cosolvents could be a critical step in improving these systems.

## Summary and Perspectives

Electrolytes play a crucial role in electrochemical devices, transporting ionic species to the electrodes and effectively determining the electrochemically stable potential window of the flexible devices. The choice of electrolytes can also have a large impact on the power and energy density of flexible supercapacitors. In this Review, the recent developments of biopolymer-based GPEs for flexible supercapacitors were summarized. It has been demonstrated that, benefiting from the unique properties of biopolymers, such as hydrophilicity, abundant functional groups, and eco-friendliness, the biopolymer-based hydrogels can serve as a promising component in high-performance devices. These bio-based polymers impart superior ionic conductivity, thermal stability, mechanical robustness, biodegradability, etc. Moreover, additional functions such as self-healing capability and wide temperature tolerance can also be integrated into biopolymer-based GPEs, establishing multifunctional supercapacitors. In this regard, the performance evaluation standards of biopolymer-based hydrogel electrolytes for supercapacitors were proposed for the first time according to the mechanism of three kinds of supercapacitors. Finally, several important issues, such as sustainability and electrode–electrolyte interfacial disconnect, and other associated challenges with hydrogel electrolytes have been discussed in detail.

**Figure 10:**
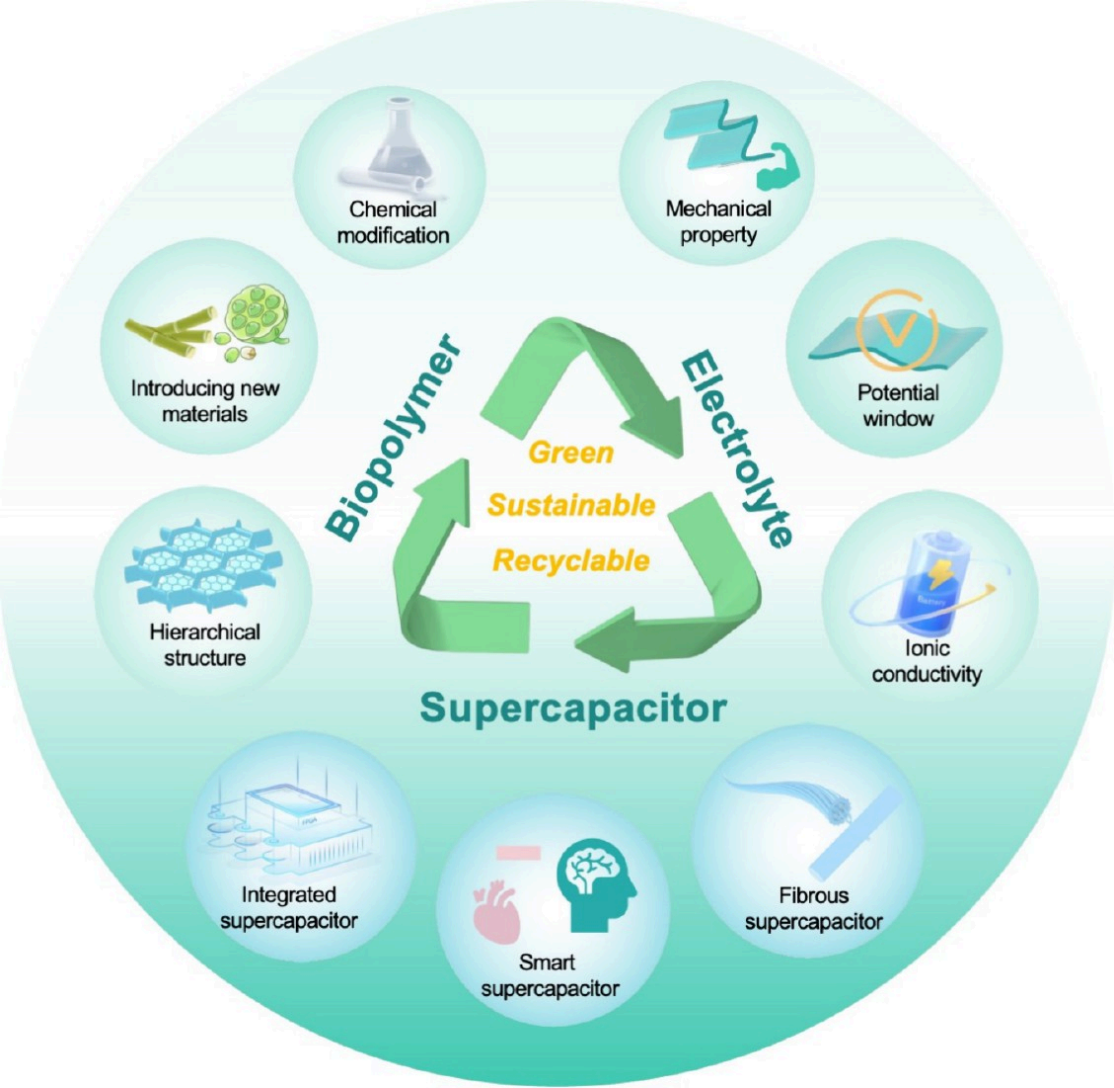
Perspectives for the future development of biopolymer-based hydrogel electrolytes for green and sustainable supercapacitors.

In spite of the tremendous achievements made with biopolymer-based GPEs in recent years, there are still some inadequacies to be improved to meet the demand in practical applications for powering various flexible and wearable electronics. Future investigation should focus on addressing the following concerns (Figure 10):

***Biopolymer*:** (1) *Hierarchical structure of biomass.* Abundant, natural, biodegradable polymers possess unique hierarchical structures. For example, some studies have reported that hierarchical and fibrous wood-based structures provide a large number of multiscale channels (e.g., lumina of tracheids, vessels, and fibers in wood as microscale channels, nanogaps between adjacent cellulose fibrils as nanoscale channels) useful for ion transport.^15^ Furthermore, the ion transport behavior can be facilely tuned by modifying the morphology (e.g., porosity, pore size, and alignment of fibrils) and the surface properties (e.g., functional groups, wettability, and surface charge),^38^ as well as the molecular structure (e.g., ion intercalation/exchange, transformation from cellulose I to cellulose II) of wood-based materials. Although many biopolymers have been developed for the hydrogel electrolyte of supercapacitors, they were only partially utilized to endow the electrolyte with distinctive properties. To this end, the inherent hierarchical structure of biomass materials that is conducive for ion transport has not been fully utilized in electrolyte materials. Therefore, more efforts should be devoted to exploring the advantages of these inherent hierarchical structures of biomass materials to construct high-performance electrolytes. (2) *Chemical modification of biopolymers.* Biopolymers, in addition to their abundance, low cost, biodegradability, and renewability, are inherently functional due to the multitude of functional groups (such as hydroxyl and ether groups) on their backbones. These functional groups impart a diversity of reactivity sites (e.g., through hydrogen bonds, electrostatic interactions, and ionic coordination). These sites enable various interactions with neighboring polymers—and ions in solvent—leading to diverse functions.^15,28^ However, these systems are also sensitive to a wide variety of factors. For instance, insufficient cross-linking reactions can cause reduced mechanical strength and thus reduced electrochemical performance.^12^ Additionally, the processing of biopolymers for practical applications in supercapacitors is limited owing to their highly ordered structure and strong inter- and intramolecular hydrogen bonding. There have been several reports of dissolution issues with regard to these biopolymer- and biomass-based systems. In particular, complex biopolymers such as cellulose often dissolve poorly in alkaline solvents and thus require very specific solution parameters in order to achieve dissolution;^135^ however, successful dissolution of biopolymers in ionic liquids has enabled vast enhancement in the design and construction of biopolymer-based ionogel electrolytes. Ionic liquids act not only as solvents but also as electrolyte components to transport ion carriers. Some ionic liquids can even act as catalysts for cross-linking reactions within the polymer networks.^136^ To enhance the hydrophilicity, dispersibility, solubility in various solvents, and interfacial compatibility with electrode components, chemical modifications such as esterification, etherification, silylation, and amination can be implemented. Furthermore, modification approaches such as carboxylation, oxidation, or nitrification can optimize the electron-transfer process through interactions with metal ions. Additionally, chemical grafting strategies, including grafting conductive polymers or nanoparticles, as well as impregnation of oxidants, can be employed to optimize ion transport. Therefore, substantial modification or chemical functionalization of biopolymers is necessary in order to enhance their ion transport properties in future works.

***Electrolyte*:** (1) *Ionic conductivity.* Various ions interact differently with biopolymer hydrogels, generating varied ionic conductivities. These interactions often occur between the electrolytic ions and the functional groups in the polymer chains. In view of electrolytic ions, previous studies have verified that the valence state, the size of the ionic radii, as well as the types of ions could significantly influence the interactions. Nevertheless, the available ion options for gel electrolytes remain relatively restricted. Future endeavors should therefore focus on investigating the diverse interactions between various salt ions and biopolymers in order to achieve enhanced ion transport and other desirable properties. Furthermore, it is imperative to consider the impact of different solvents and biomass materials, such as aqueous electrolytes, which can influence the solubility, hydration, degradation, crystallinity, and mechanical characteristics. By comprehensively analyzing these factors, biomass-based electrolyte systems can be further understood, and they can achieve their full potential. In terms of biopolymers, the molecular weight, the length, and the side chain groups of polymer chains will also affect the interactions. For example, hydrophilic groups on the biopolymers could facilitate the “salting in” of ions. Additionally, the degree of cross-linking among polymer chains is also an important factor for ionic conductivity. In the future, there is an opportunity to investigate the alterations in cross-linking properties of biopolymer-based hydrogels by pretreatment techniques (e.g., prestretching or cyclic dehydration), which can greatly influence ion conduction processes. It is intriguing to observe how novel treatment design strategies will influence biopolymer-based hydrogels, ultimately resulting in enhanced electrochemical performance. (2) *Electrochemically stable potential window.* As is well known, the aqueous solution within a hydrogel is a critical component and impacts the ionic conductivity of the electrolyte. However, considering that the electrochemical reactions are constrained by water-splitting reactions, i.e., the oxygen-evolution reaction and the hydrogen-evolution reaction, supercapacitors utilizing aqueous hydrogel electrolytes usually have an open-circuit voltage of between 0.8 and 2.0 V, which is much lower than that of an organic or ionic liquid electrolyte. Therefore, the practical applications of hydrogel gel electrolytes in supercapacitors are limited by their voltage and energy density. Although some pioneering works have proposed the novel “water-in-salt” electrolyte with a broad potential window where the high concentrations of salt can effectively suppress the decomposition of water, insufficient attention has been paid to the biopolymer-based hydrogel electrolytes with a wide potential window. The crucial factor in expanding the voltage window within aqueous electrolyte lies in reducing the reactivity of water. Thus, it is necessary to control humidity levels during pretreatment in order to restrict the mobility of bulk water and strengthen the binding between biopolymers and free water. Enhancing the cohesive properties of interfacial water in biomass materials represents a pivotal challenge that must be addressed in the foreseeable future. In addition, the inevitable evaporation of water within hydrogels leads to diminished ionic conductivity and cycling stability. Certainly, this issue can be addressed through introducing species with strong interactions with water (i.e., hydration of lithium ions). Overall, aqueous solutions inside the biopolymer-based hydrogel electrolyte need to be modulated to achieve the desired potential window and ionic conductivity.

***Supercapacitor*:** The extraordinary characteristics exhibited by biopolymers have imbued supercapacitors with renewed vigor, but a substantial gap remains in the exploration and implementation of these materials in this domain. In the future, the exploration of biopolymer-based supercapacitors should focus on the following concepts. First, the rapid development of small-sized and portable electronic equipment causes tremendous pressure in the demand for diverse geometries or configurations of biopolymer-based supercapacitors. Accordingly, sandwich-type, fiber-shaped, and interdigitated designs can also be presented to meet the demands of portability and wearability. Second, the versatility of biopolymer-based hydrogel electrolytes has propelled the advancement of multifunctional supercapacitors as independent power supplies, including stretchable, smart-responsive, self-healing, and shape-memory supercapacitors. Finally, integrated supercapacitors can be combined with energy harvesters (e.g., photovoltaic, triboelectric, and piezoelectric) or sensors to generate self-powered devices and supercapacitor-driven sensor systems. For instance, integrating biodegradable supercapacitors with sensing devices holds immense potential in shaping integrated circuits dedicated to non-polluting environmental assessments and human physiological testing, among many other fascinating features. All in all, the superior properties of biopolymers would inject new vitality into the future development of green and sustainable supercapacitors that could aid in meeting the diverse demands of humans.

## References

[ref1] Li M.; Zhou S.; Cheng L.; Mo F.; Chen L.; Yu S.; Wei J. 3D Printed Supercapacitor: Techniques, Materials, Designs, and Applications. Adv. Funct. Mater. 2023, 33 (1), 2208034 10.1002/adfm.202208034.

[ref2] Heligman B. T.; Manthiram A. Elemental Foil Anodes for Lithium-Ion Batteries. ACS Energy Lett. 2021, 6 (8), 2666–2672. 10.1021/acsenergylett.1c01145.

[ref3] Wang Z.; Li H.; Tang Z.; Liu Z.; Ruan Z.; Ma L.; Yang Q.; Wang D.; Zhi C. Hydrogel Electrolytes for Flexible Aqueous Energy Storage Devices. Adv. Funct. Mater. 2018, 28 (48), 1804560 10.1002/adfm.201804560.

[ref4] Zhong C.; Deng Y.; Hu W.; Qiao J.; Zhang L.; Zhang J. A review of electrolyte materials and compositions for electrochemical supercapacitors. Chem. Soc. Rev. 2015, 44 (21), 7484–7539. 10.1039/C5CS00303B. 26050756

[ref5] Chan C. Y.; Wang Z.; Jia H.; Ng P. F.; Chow L.; Fei B. Recent advances of hydrogel electrolytes in flexible energy storage devices. J. Mater. Chem. A 2021, 9 (4), 2043–2069. 10.1039/D0TA09500A.

[ref6] Qiu Z.; Shi L.; Wang Z.; Mindemark J.; Zhu J.; Edström K.; Zhao Y.; Yuan S. Surface activated polyethylene separator promoting Li+ ion transport in gel polymer electrolytes and cycling stability of Li-metal anode. Chem. Eng. J. 2019, 368, 321–330. 10.1016/j.cej.2019.02.107.

[ref7] Yan J.; Liu F.-Q.; Gao J.; Zhou W.; Huo H.; Zhou J.-J.; Li L. Low-Cost Regulating Lithium Deposition Behaviors by Transition Metal Oxide Coating on Separator. Adv. Funct. Mater. 2021, 31 (16), 2007255 10.1002/adfm.202007255.

[ref8] Chen W.-J.; Li B.-Q.; Zhao C.-X.; Zhao M.; Yuan T.-Q.; Sun R.-C.; Huang J.-Q.; Zhang Q. Electrolyte Regulation towards Stable Lithium-Metal Anodes in Lithium-Sulfur Batteries with Sulfurized Polyacrylonitrile Cathodes. Angew. Chem., Int. Ed. 2020, 59 (27), 10732–10745. 10.1002/anie.201912701. 31746521

[ref9] Wang C.; Yokota T.; Someya T. Natural Biopolymer-Based Biocompatible Conductors for Stretchable Bioelectronics. Chem. Rev. 2021, 121 (4), 2109–2146. 10.1021/acs.chemrev.0c00897. 33460327

[ref10] Fan X.; Zhong C.; Liu J.; Ding J.; Deng Y.; Han X.; Zhang L.; Hu W.; Wilkinson D. P.; Zhang J. Opportunities of Flexible and Portable Electrochemical Devices for Energy Storage: Expanding the Spotlight onto Semi-solid/Solid Electrolytes. Chem. Rev. 2022, 122 (23), 17155–17239. 10.1021/acs.chemrev.2c00196. 36239919

[ref11] Wu L.; Shi X.; Wu Z. S. Recent Advancements and Perspectives of Biodegradable Polymers for Supercapacitors. Adv. Funct. Mater. 2023, 33 (16), 2211454 10.1002/adfm.202211454.

[ref12] Xu T.; Liu K.; Sheng N.; Zhang M.; Liu W.; Liu H.; Dai L.; Zhang X.; Si C.; Du H.; Zhang K. Biopolymer-based hydrogel electrolytes for advanced energy storage/conversion devices: properties, applications, and perspectives. Energy Storage Mater. 2022, 48, 244–262. 10.1016/j.ensm.2022.03.013.

[ref13] D’Acierno F.; Michal C. A.; MacLachlan M. J. Thermal Stability of Cellulose Nanomaterials. Chem. Rev. 2023, 123 (11), 7295–7325. 10.1021/acs.chemrev.2c00816. 37132652

[ref14] Chen W.; Yu H.; Lee S. Y.; Wei T.; Li J.; Fan Z. Nanocellulose: a promising nanomaterial for advanced electrochemical energy storage. Chem. Soc. Rev. 2018, 47 (8), 2837–2872. 10.1039/C7CS00790F. 29561005

[ref15] Chen C.; Hu L. Nanoscale Ion Regulation in Wood-Based Structures and Their Device Applications. Adv. Mater. 2021, 33 (28), e2002890 10.1002/adma.202002890. 33108027

[ref16] Foster E. J.; Moon R. J.; Agarwal U. P.; Bortner M. J.; Bras J.; Camarero-Espinosa S.; Chan K. J.; Clift M. J. D.; Cranston E. D.; Eichhorn S. J.; et al. Current characterization methods for cellulose nanomaterials. Chem. Soc. Rev. 2018, 47 (8), 2609–2679. 10.1039/C6CS00895J. 29658545

[ref17] Thongsomboon W.; Serra D. O.; Possling A.; Hadjineophytou C.; Hengge R.; Cegelski L. Phosphoethanolamine cellulose: A naturally produced chemically modified cellulose. Science 2018, 359 (6373), 334–338. 10.1126/science.aao4096. 29348238

[ref18] Adler A.; Kumaniaev I.; Karacic A.; Baddigam K. R.; Hanes R. J.; Subbotina E.; Bartling A. W.; Huertas-Alonso A. J.; Moreno A.; Håkansson H.; et al. Lignin-first biorefining of Nordic poplar to produce cellulose fibers could displace cotton production on agricultural lands. Joule 2022, 6 (8), 1845–1858. 10.1016/j.joule.2022.06.021.

[ref19] Gregory D. A.; Tripathi L.; Fricker A. T. R.; Asare E.; Orlando I.; Raghavendran V.; Roy I. Bacterial cellulose: A smart biomaterial with diverse applications. Mater. Sci. Eng.: R: Reports 2021, 145, 100623 10.1016/j.mser.2021.100623.

[ref20] Koivikko A.; Lampinen V.; Yiannacou K.; Sharma V.; Sariola V. Biodegradable, Flexible and Transparent Tactile Pressure Sensor Based on Rubber Leaf Skeletons. IEEE Sensors J. 2022, 22 (12), 11241–11247. 10.1109/JSEN.2021.3078807.

[ref21] Nakamura K.; Kubota R.; Aoyama T.; Urayama K.; Hamachi I. Four distinct network patterns of supramolecular/polymer composite hydrogels controlled by formation kinetics and interfiber interactions. Nat. Commun. 2023, 14 (1), 1696 10.1038/s41467-023-37412-0. 36973291 PMC10042874

[ref22] Liu W.; Liu K.; Du H.; Zheng T.; Zhang N.; Xu T.; Pang B.; Zhang X.; Si C.; Zhang K. Cellulose Nanopaper: Fabrication, Functionalization, and Applications. Nano-Micro Lett. 2022, 14 (104), 820–849. 10.1007/s40820-022-00849-x. PMC9008119 35416525

[ref23] Qiang H.; He W.; Guo F.; Cao J.; Wang R.; Guo Z. Layer-by-Layer Self-Assembled TEMPO-Oxidized Cellulose Nanofiber/Reduced Graphene Oxide/Polypyrrole Films for Self-Supporting Flexible Supercapacitor Electrodes. ACS Appl. Nano Mater. 2022, 5 (5), 6305–6315. 10.1021/acsanm.2c00397.

[ref24] Ye Y.; Oguzlu H.; Zhu J.; Zhu P.; Yang P.; Zhu Y.; Wan Z.; Rojas O. J.; Jiang F. Ultrastretchable Ionogel with Extreme Environmental Resilience through Controlled Hydration Interactions. Adv. Funct. Mater. 2023, 33 (2), 2209787 10.1002/adfm.202209787.

[ref25] Fang Z.; Flynn M. G.; Jackson J. E.; Hegg E. L. Thio-assisted reductive electrolytic cleavage of lignin β-O-4 models and authentic lignin. Green Chem. 2021, 23 (1), 412–421. 10.1039/D0GC03597A.

[ref26] Peng H.; Gao X.; Sun K.; Xie X.; Ma G.; Zhou X.; Lei Z. Physically cross-linked dual-network hydrogel electrolyte with high self-healing behavior and mechanical strength for wide-temperature tolerant flexible supercapacitor. Chem. Eng. J. 2021, 422, 130353 10.1016/j.cej.2021.130353.

[ref27] Wang J.; Gao C.; Hou P.; Liu Y.; Zhao J.; Huo P. All-bio-based, adhesive and low-temperature resistant hydrogel electrolytes for flexible supercapacitors. Chem. Eng. J. 2023, 455, 140952 10.1016/j.cej.2022.140952.

[ref28] Ye Y.; Yu L.; Lizundia E.; Zhu Y.; Chen C.; Jiang F. Cellulose-Based Ionic Conductor: An Emerging Material toward Sustainable Devices. Chem. Rev. 2023, 123 (15), 9204–9264. 10.1021/acs.chemrev.2c00618. 37419504

[ref29] Quan Y.; Zhou W.; Wu T.; Chen M.; Han X.; Tian Q.; Xu J.; Chen J. Sorbitol-modified cellulose hydrogel electrolyte derived from wheat straws towards high-performance environmentally adaptive flexible zinc-ion batteries. Chem. Eng. J. 2022, 446, 137056 10.1016/j.cej.2022.137056.

[ref30] Sheng O.; Jin C.; Yang T.; Ju Z.; Luo J.; Tao X. Designing biomass-integrated solid polymer electrolytes for safe and energy-dense lithium metal batteries. Energy Environ. Sci. 2023, 16 (7), 2804–2824. 10.1039/D3EE01173A.

[ref31] Zhang Q.; Zhao L.; Yang H.; Kong L.; Ran F. Alkali-tolerant polymeric gel electrolyte membrane based on cross-linked carboxylated chitosan for supercapacitors. J. Membr. Sci. 2021, 629, 119083 10.1016/j.memsci.2021.119083.

[ref32] Yang H.; Ji X.; Tan Y.; Liu Y.; Ran F. Modified supramolecular carboxylated chitosan as hydrogel electrolyte for quasi-solid-state supercapacitors. J. Power Sources 2019, 441, 227174 10.1016/j.jpowsour.2019.227174.

[ref33] Zhang K.; Pang Y.; Chen C.; Wu M.; Liu Y.; Yu S.; Li L.; Ji Z.; Pang J. Stretchable and conductive cellulose hydrogel electrolytes for flexible and foldable solid-state supercapacitors. Carbohydr. Polym. 2022, 293, 119673 10.1016/j.carbpol.2022.119673. 35798414

[ref34] Lin C.-H.; Wang P.-H.; Lee W.-N.; Li W.-C.; Wen T.-C. Chitosan with various degrees of carboxylation as hydrogel electrolyte for pseudo solid-state supercapacitors. J. Power Sources 2021, 494, 229736 10.1016/j.jpowsour.2021.229736.

[ref35] Chen J.; Yu Q.; Shi D.; Yang Z.; Dong K.; Kaneko D.; Dong W.; Chen M. Tough and Antifreezing Organohydrogel Electrolyte for Flexible Supercapacitors with Wide Temperature Stability. ACS Appl. Energy Mater. 2021, 4 (9), 9353–9361. 10.1021/acsaem.1c01556.

[ref36] Wang D.; Li Z.; Yang L.; Zhang J.; Wei Y.; Feng Q.; Wei Q. Hydrogel electrolyte based on sodium polyacrylate/KOH hydrogel reinforced with bacterial cellulose aerogel for flexible supercapacitors. Chem. Eng. J. 2023, 454, 140090 10.1016/j.cej.2022.140090.

[ref37] Qiu F.; Huang Y.; He G.; Luo C.; Li X.; Wang M.; Wu Y. A lignocellulose-based neutral hydrogel electrolyte for high-voltage supercapacitors with overlong cyclic stability. Electrochim. Acta 2020, 363, 137241 10.1016/j.electacta.2020.137241.

[ref38] Jing S.; Wu L.; Siciliano A. P.; Chen C.; Li T.; Hu L. The Critical Roles of Water in the Processing, Structure, and Properties of Nanocellulose. ACS Nano 2023, 17 (22), 22196–22226. 10.1021/acsnano.3c06773. 37934794

[ref39] Gao C.; Gao Z.; Wei Y.; Luo N.; Liu Y.; Huo P. Flexible Wood Enhanced Poly(acrylic acid-co-acrylamide)/Quaternized Gelatin Hydrogel Electrolytes for High-Energy-Density Supercapacitors. ACS Appl. Mater. Interfaces 2023, 15 (2), 2951–2960. 10.1021/acsami.2c18935. 36597008

[ref40] Alipoori S.; Mazinani S.; Aboutalebi S. H.; Sharif F. Review of PVA-based gel polymer electrolytes in flexible solid-state supercapacitors: Opportunities and challenges. J. Energy Storage 2020, 27, 101072 10.1016/j.est.2019.101072.

[ref41] An T.; Cheng W. Recent progress in stretchable supercapacitors. J. Mater. Chem. A 2018, 6 (32), 15478–15494. 10.1039/C8TA03988G.

[ref42] Huang J.; Xie Y.; You Y.; Yuan J.; Xu Q.; Xie H.; Chen Y. Rational Design of Electrode Materials for Advanced Supercapacitors: From Lab Research to Commercialization. Adv. Funct. Mater. 2023, 33 (14), 2213095 10.1002/adfm.202213095.

[ref43] Choudhary N.; Li C.; Moore J.; Nagaiah N.; Zhai L.; Jung Y.; Thomas J. Asymmetric supercapacitor electrodes and devices. Adv. Mater. 2017, 29 (21), 1605336 10.1002/adma.201605336. 28244158

[ref44] González A.; Goikolea E.; Barrena J. A.; Mysyk R. Review on supercapacitors: Technologies and materials. Renew. Sust. Energy Rev. 2016, 58, 1189–1206. 10.1016/j.rser.2015.12.249.

[ref45] Bhat T. S.; Patil P. S.; Rakhi R. B. Recent trends in electrolytes for supercapacitors. J. Energy Storage 2022, 50, 104222 10.1016/j.est.2022.104222.

[ref46] Liu T.-C.; Sutarsis S.; Zhong X.-Y.; Lin W.-C.; Chou S.-H.; Kirana N.; Huang P.-Y.; Lo Y.-C.; Chang J.-K.; Wu P.-W.; et al. An interfacial wetting water based hydrogel electrolyte for high-voltage flexible quasi solid-state supercapacitors. Energy Storage Mater. 2021, 38, 489–498. 10.1016/j.ensm.2021.03.028.

[ref47] Balasubramaniam S.; Mohanty A.; Balasingam S. K.; Kim S. J.; Ramadoss A. Comprehensive Insight into the Mechanism, Material Selection and Performance Evaluation of Supercapatteries. Nano-Micro Lett. 2020, 12, 85 10.1007/s40820-020-0413-7. PMC7770895 34138304

[ref48] Li H.; Qi C.; Tao Y.; Liu H.; Wang D. W.; Li F.; Yang Q. H.; Cheng H. M. Quantifying the Volumetric Performance Metrics of Supercapacitors. Adv. Energy Mater. 2019, 9 (21), 1900079 10.1002/aenm.201900079.

[ref49] Raravikar N.; Dobos A.; Narayanan E.; Grandhi T. S. P.; Mishra S.; Rege K.; Goryll M. Investigation into Pseudo-Capacitance Behavior of Glycoside-Containing Hydrogels. ACS Appl. Mater. Interfaces 2017, 9 (4), 3554–3561. 10.1021/acsami.6b11113. 28067487

[ref50] Koudriachova M. V.; Harrison N. M.; de Leeuw S. W. Effect of diffusion on lithium intercalation in titanium dioxide. Phys. Rev. Lett. 2001, 86 (7), 1275 10.1103/PhysRevLett.86.1275. 11178062

[ref51] Wu Z.; Wang B.; Li J.; Wu R.; Jin M.; Zhao H.; Chen S.; Wang H. Advanced Bacterial Cellulose Ionic Conductors with Gigantic Thermopower for Low-Grade Heat Harvesting. Nano Lett. 2022, 22 (20), 8152–8160. 10.1021/acs.nanolett.2c02558. 36219168

[ref52] Tian C.; Wang J.; Sun R.; Ali T.; Wang H.; Xie B.-B.; Zhong Y.; Hu Y. Improved Interfacial Ion Migration and Deposition through the Chain-Liquid Synergistic Effect by a Carboxylated Hydrogel Electrolyte for Stable Zinc Metal Anodes. Angew. Chem., Int. Ed. 2023, 62, e202310970 10.1002/anie.202310970. 37644643

[ref53] Sun W.; Xu Z.; Qiao C.; Lv B.; Gai L.; Ji X.; Jiang H.; Liu L. Antifreezing Proton Zwitterionic Hydrogel Electrolyte via Ionic Hopping and Grotthuss Transport Mechanism toward Solid Supercapacitor Working at −50°C. Adv. Sci. 2022, 9 (27), 2201679 10.1002/advs.202201679. PMC9507348 35882629

[ref54] Cheng X.; Pan J.; Zhao Y.; Liao M.; Peng H. Gel Polymer Electrolytes for Electrochemical Energy Storage. Adv. Energy Mater. 2018, 8 (7), 1702184 10.1002/aenm.201702184.

[ref55] Zhang S.; Pan N. Supercapacitors performance evaluation. Adv. Energy Mater. 2015, 5 (6), 1401401 10.1002/aenm.201401401.

[ref56] Noori A.; El-Kady M. F.; Rahmanifar M. S.; Kaner R. B.; Mousavi M. F. Towards establishing standard performance metrics for batteries, supercapacitors and beyond. Chem. Soc. Rev. 2019, 48 (5), 1272–1341. 10.1039/C8CS00581H. 30741286

[ref57] Zhao J.; Burke A. F. Electrochemical capacitors: performance metrics and evaluation by testing and analysis. Adv. Energy Mater. 2021, 11 (1), 2002192 10.1002/aenm.202002192.

[ref58] Lukatskaya M. R.; Dunn B.; Gogotsi Y. Multidimensional materials and device architectures for future hybrid energy storage. Nat. Commun. 2016, 7, 12647 10.1038/ncomms12647. 27600869 PMC5023960

[ref59] Zhou C.; Lin H.; He Q.; Xu L.; Worku M.; Chaaban M.; Lee S.; Shi X.; Du M.-H.; Ma B. Low dimensional metal halide perovskites and hybrids. Mater. Sci. Eng.: R: Reports 2019, 137, 38–65. 10.1016/j.mser.2018.12.001.

[ref60] Yan L.; Huang J.; Dong X.; Guo Z.; Wang Z.; Wang Y. Energizing hybrid supercapacitors by using Mn^2+^-based active electrolyte. J. Mater. Chem. A 2020, 8 (30), 15051–15057. 10.1039/D0TA04864J.

[ref61] Burke A.; Miller M. Testing of electrochemical capacitors: Capacitance, resistance, energy density, and power capability. Electrochim. Acta 2010, 55 (25), 7538–7548. 10.1016/j.electacta.2010.04.074.

[ref62] Augustyn V.; Simon P.; Dunn B. Pseudocapacitive oxide materials for high-rate electrochemical energy storage. Energy Environ. Sci. 2014, 7 (5), 1597 10.1039/c3ee44164d.

[ref63] Miller J. R.; Outlaw R.; Holloway B. Graphene double-layer capacitor with ac line-filtering performance. Science 2010, 329 (5999), 1637–1639. 10.1126/science.1194372. 20929845

[ref64] Li Z.; Li L.; Li Z.; Liao H.; Zhang H. Ultrathin carbon gauze for high-rate supercapacitor. Electrochim. Acta 2016, 222, 990–998. 10.1016/j.electacta.2016.11.067.

[ref65] Conway B. E. Transition from ‘supercapacitor’ to ‘battery’ behavior in electrochemical energy storage. Proc. 34th Int. Power Sources Symp. 1990, 138, 319–327. 10.1109/IPSS.1990.145856.

[ref66] Shodiev A.; Zanotto F. M.; Yu J.; Chouchane M.; Li J.; Franco A. A. Designing electrode architectures to facilitate electrolyte infiltration for lithium-ion batteries. Energy Storage Mater. 2022, 49, 268–277. 10.1016/j.ensm.2022.03.049.

[ref67] Wang Z.; Heasman P.; Rostami J.; Benselfelt T.; Linares M.; Li H.; Iakunkov A.; Sellman F.; Östmans R.; Hamedi M. M.; et al. Dynamic Networks of Cellulose Nanofibrils Enable Highly Conductive and Strong Polymer Gel Electrolytes for Lithium-Ion Batteries. Adv. Funct. Mater. 2023, 33 (30), 2212806 10.1002/adfm.202212806.

[ref68] Zhang L.; Wang S.; Wang Z.; Liu Z.; Xu X.; Liu H.; Wang D.; Tian Z. Temperature-Mediated Phase Separation Enables Strong yet Reversible Mechanical and Adhesive Hydrogels. ACS Nano 2023, 17 (14), 13948–13960. 10.1021/acsnano.3c03910. 37428219

[ref69] Lu J.; Lin X.; Wang S.; Xu X.; Zhou Y.; Zhang Y.; Li Q.; Liu H. High ionic conductivity and toughness hydrogel electrolyte for high-performance flexible solid-state zinc-ion hybrid supercapacitors enabled by cellulose-bentonite coordination interactions. Green Chem. 2023, 25 (4), 1635–1646. 10.1039/D2GC04711J.

[ref70] Li Y.; Gong Q.; Liu X.; Xia Z.; Yang Y.; Chen C.; Qian C. Wide temperature-tolerant polyaniline/cellulose/polyacrylamide hydrogels for high-performance supercapacitors and motion sensors. Carbohydr. Polym. 2021, 267, 118207 10.1016/j.carbpol.2021.118207. 34119166

[ref71] Park J. H.; Rana H. H.; Lee J. Y.; Park H. S. Renewable flexible supercapacitors based on all-lignin-based hydrogel electrolytes and nanofiber electrodes. J. Mater. Chem. A 2019, 7 (28), 16962–16968. 10.1039/C9TA03519B.

[ref72] Mahfoudhi N.; Boufi S. Poly (acrylic acid-co-acrylamide)/cellulose nanofibrils nanocomposite hydrogels: effects of CNFs content on the hydrogel properties. Cellulose 2016, 23 (6), 3691–3701. 10.1007/s10570-016-1074-z.

[ref73] Lin X.; Wang M.; Zhao J.; Wu X.; Xie J.; Yang J. Super-tough and self-healable all-cellulose-based electrolyte for fast degradable quasi-solid-state supercapacitor. Carbohydr. Polym. 2023, 304, 120502 10.1016/j.carbpol.2022.120502. 36641192

[ref74] Dai L.; Arcelus O.; Sun L.; Wang H.; Carrasco J.; Zhang H.; Zhang W.; Tang J. Embedded 3D Li^+^ channels in a water-in-salt electrolyte to develop flexible supercapacitors and lithium-ion batteries. J. Mater. Chem. A 2019, 7 (43), 24800–24806. 10.1039/C9TA08699D.

[ref75] Zhang Q.; Hou X.; Liu X.; Xie X.; Duan L.; Lu W.; Gao G. Nucleotide-Tackified Organohydrogel Electrolyte for Environmentally Self-Adaptive Flexible Supercapacitor with Robust Electrolyte/Electrode Interface. Small 2021, 17 (46), e2103091 10.1002/smll.202103091. 34643034

[ref76] Xu J.; Jin R.; Ren X.; Gao G. A wide temperature-tolerant hydrogel electrolyte mediated by phosphoric acid towards flexible supercapacitors. Chem. Eng. J. 2021, 413, 127446 10.1016/j.cej.2020.127446.

[ref77] Hu O.; Lu J.; Chen G.; Chen K.; Gu J.; Weng S.; Hou L.; Zhang X.; Jiang X. An Antifreezing, Tough, Rehydratable, and Thermoplastic Poly(vinyl alcohol)/Sodium Alginate/Poly(ethylene glycol) Organohydrogel Electrolyte for Flexible Supercapacitors. ACS Sustainable Chem. Eng. 2021, 9 (29), 9833–9845. 10.1021/acssuschemeng.1c02464.

[ref78] Wu S.; Lou D.; Wang H.; Jiang D.; Fang X.; Meng J.; Sun X.; Li J. One-pot synthesis of anti-freezing carrageenan/polyacrylamide double-network hydrogel electrolyte for low-temperature flexible supercapacitors. Chem. Eng. J. 2022, 435, 135057 10.1016/j.cej.2022.135057.

[ref79] Lin T.; Shi M.; Huang F.; Peng J.; Bai Q.; Li J.; Zhai M. One-Pot Synthesis of a Double-Network Hydrogel Electrolyte with Extraordinarily Excellent Mechanical Properties for a Highly Compressible and Bendable Flexible Supercapacitor. ACS Appl. Mater. Interfaces 2018, 10 (35), 29684–29693. 10.1021/acsami.8b11377. 30088910

[ref80] Cevik E.; Gunday S. T.; Bozkurt A.; Iqbal A.; Asiri S. M.; Alqarni A. N.; Almofleh A. Scalable, Quasi-Solid-State Bio-polymer Hydrogel Electrolytes for High-Performance Supercapacitor Applications. ACS Sustainable Chem. Eng. 2022, 10 (33), 10839–10848. 10.1021/acssuschemeng.2c02281.

[ref81] Xu C.; Guan S.; Dong X.; Qi M. Super strong gelatin/cellulose nanofiber hybrid hydrogels without covalent cross-linking for strain sensor and supercapacitor. Composites A: Appl. Sci. Manuf. 2023, 164, 107287 10.1016/j.compositesa.2022.107287.

[ref82] Wang D.; Yang F.; Cong L.; Feng W.; Wang C.; Chu F.; Nan J.; Chen R. Lignin-containing hydrogel matrices with enhanced adhesion and toughness for all-hydrogel supercapacitors. Chem. Eng. J. 2022, 450, 138025 10.1016/j.cej.2022.138025.

[ref83] Nan J.; Zhang G.; Zhu T.; Wang Z.; Wang L.; Wang H.; Chu F.; Wang C.; Tang C. A Highly Elastic and Fatigue-Resistant Natural Protein-Reinforced Hydrogel Electrolyte for Reversible-Compressible Quasi-Solid-State Supercapacitors. Adv. Sci. 2020, 7 (14), 2000587 10.1002/advs.202000587. PMC7375230 32714764

[ref84] Wang Y.; Chen F.; Liu Z.; Tang Z.; Yang Q.; Zhao Y.; Du S.; Chen Q.; Zhi C. A Highly Elastic and Reversibly Stretchable All-Polymer Supercapacitor. Angew. Chem., Int. Ed. 2019, 58 (44), 15707–15711. 10.1002/anie.201908985. 31441591

[ref85] Liu T.; Ren X.; Zhang J.; Liu J.; Ou R.; Guo C.; Yu X.; Wang Q.; Liu Z. Highly compressible lignin hydrogel electrolytes via double-crosslinked strategy for superior foldable supercapacitors. J. Power Sources 2020, 449, 227532 10.1016/j.jpowsour.2019.227532.

[ref86] Zhao J.; Chen Y.; Yao Y.; Tong Z.-R.; Li P.-W.; Yang Z.-M.; Jin S.-H. Preparation of the polyelectrolyte complex hydrogel of biopolymers via a semi-dissolution acidification sol-gel transition method and its application in solid-state supercapacitors. J. Power Sources 2018, 378, 603–609. 10.1016/j.jpowsour.2018.01.005.

[ref87] Peng K.; Zhang J.; Yang J.; Lin L.; Gan Q.; Yang Z.; Chen Y.; Feng C. Green Conductive Hydrogel Electrolyte with Self-Healing Ability and Temperature Adaptability for Flexible Supercapacitors. ACS Appl. Mater. Interfaces 2022, 14 (34), 39404–39419. 10.1021/acsami.2c11973. 35981091

[ref88] Peng Z.; Zou Y.; Xu S.; Zhong W.; Yang W. High-Performance Biomass-Based Flexible Solid-State Supercapacitor Constructed of Pressure-Sensitive Lignin-Based and Cellulose Hydrogels. ACS Appl. Mater. Interfaces 2018, 10 (26), 22190–22200. 10.1021/acsami.8b05171. 29882652

[ref89] Zhu X.; Ji C.; Meng Q.; Mi H.; Yang Q.; Li Z.; Yang N.; Qiu J. Freeze-tolerant hydrogel electrolyte with high strength for stable operation of flexible zinc-ion hybrid supercapacitors. Small 2022, 18 (16), 2200055 10.1002/smll.202200055. 35274442

[ref90] Yun T. G.; Jang J.-S.; Cheong J. Y.; Kim I.-D. Organism epidermis/plant-root inspired ultra-stable supercapacitor for large-scale wearable energy storage applications. Nano Energy 2021, 82, 105776 10.1016/j.nanoen.2021.105776.

[ref91] Deng Y.; Wang H.; Zhang K.; Qiu J.; Yan L. Flexible Quasi-Solid-State High-Performance Aqueous Zinc Ion Hybrid Supercapacitor with Water-in-Salt Hydrogel Electrolyte and N/P-Dual Doped Graphene Hydrogel Electrodes. Adv. Sust. Syst. 2022, 6 (1), 2100191 10.1002/adsu.202100191.

[ref92] Li X.; Yuan L.; Liu R.; He H.; Hao J.; Lu Y.; Wang Y.; Liang G.; Yuan G.; Guo Z. Engineering textile electrode and bacterial cellulose nanofiber reinforced hydrogel electrolyte to enable high-performance flexible all-solid-state supercapacitors. Adv. Energy Mater. 2021, 11 (12), 2003010 10.1002/aenm.202003010.

[ref93] Zeng J.; Dong L.; Sha W.; Wei L.; Guo X. Highly stretchable, compressible and arbitrarily deformable all-hydrogel soft supercapacitors. Chem. Eng. J. 2020, 383, 123098 10.1016/j.cej.2019.123098.

[ref94] Deng Y.; Wang H.; Zhang K.; Shao J.; Qiu J.; Wu J.; Wu Y.; Yan L. A high-voltage quasi-solid-state flexible supercapacitor with a wide operational temperature range based on a low-cost ″water-in-salt″ hydrogel electrolyte. Nanoscale 2021, 13 (5), 3010–3018. 10.1039/D0NR08437A. 33508053

[ref95] Wang H.; Deng Y.; Qiu J.; Wu J.; Zhang K.; Shao J.; Yan L. In Situ Formation of ″Dimethyl Sulfoxide/Water-in-Salt″-Based Chitosan Hydrogel Electrolyte for Advanced All-Solid-State Supercapacitors. ChemSusChem 2021, 14 (2), 632–641. 10.1002/cssc.202002236. 33047843

[ref96] Huang H.; Han L.; Fu X.; Wang Y.; Yang Z.; Pan L.; Xu M. A Powder Self-Healable Hydrogel Electrolyte for Flexible Hybrid Supercapacitors with High Energy Density and Sustainability. Small 2021, 17 (10), e2006807 10.1002/smll.202006807. 33590690

[ref97] Chen M.; Chen J.; Zhou W.; Xu J.; Wong C.-P. High-performance flexible and self-healable quasi-solid-state zinc-ion hybrid supercapacitor based on borax-crosslinked polyvinyl alcohol/nanocellulose hydrogel electrolyte. J. Mater. Chem. A 2019, 7 (46), 26524–26532. 10.1039/C9TA10944G.

[ref98] Wang H.; Wu J.; Qiu J.; Zhang K.; Shao J.; Yan L. In Situ Formation of Renewable Cellulose Hydrogel Electrolyte for High-performance Flexible All-Solid-State Asymmetric Supercapacitors. Sustainable Energy Fuels 2019, 3 (33), 3109–3115. 10.1039/C9SE00339H.

[ref99] Zhao W.; Wei L.; Fu Q.; Guo X. High-performance, flexible, solid-state micro-supercapacitors based on printed asymmetric interdigital electrodes and bio-hydrogel for on-chip electronics. J. Power Sources 2019, 422, 73–83. 10.1016/j.jpowsour.2019.03.021.

[ref100] Xu T.; Du H.; Liu H.; Liu W.; Zhang X.; Si C.; Liu P.; Zhang K. Advanced Nanocellulose-Based Composites for Flexible Functional Energy Storage Devices. Adv. Mater. 2021, 33 (48), 2101368 10.1002/adma.202101368. PMC11468700 34561914

[ref101] Cao G.; Zhao L.; Ji X.; Peng Y.; Yu M.; Wang X.; Li X.; Ran F. ″Salting out″ in Hofmeister Effect Enhancing Mechanical and Electrochemical Performance of Amide-based Hydrogel Electrolytes for Flexible Zinc-Ion Battery. Small 2023, 19 (30), e2207610 10.1002/smll.202207610. 37026666

[ref102] Liu Z.; Liang G.; Zhan Y.; Li H.; Wang Z.; Ma L.; Wang Y.; Niu X.; Zhi C. A soft yet device-level dynamically super-tough supercapacitor enabled by an energy-dissipative dual-crosslinked hydrogel electrolyte. Nano Energy 2019, 58, 732–742. 10.1016/j.nanoen.2019.01.087.

[ref103] Yuan N.; Xu L.; Xu B.; Zhao J.; Rong J. Chitosan derivative-based self-healable hydrogels with enhanced mechanical properties by high-density dynamic ionic interactions. Carbohydr. Polym. 2018, 193, 259–267. 10.1016/j.carbpol.2018.03.071. 29773380

[ref104] Wang Y.; Jiang W.; Li J.; Ahommed M. S.; Wang C.; Ji X.; Liu Y.; Yang G.; Ni Y.; Lyu G. Zinc-ion engineered Plant-based multifunctional hydrogels for flexible wearable strain Sensors, Bio-electrodes and Zinc-ion hybrid capacitors. Chem. Eng. J. 2023, 465, 142917 10.1016/j.cej.2023.142917.

[ref105] Wang X.; Xing Z.; Yang C.; Qin Y. A Stretchable and Healable Gelatin Hydrogel Assisted by Hofmeister Effect for All-in-One Flexible Supercapacitor. Energy Technol. 2022, 10 (12), 2200897 10.1002/ente.202200897.

[ref106] Zhang Y.; Cremer P. S. Interactions between macromolecules and ions: The Hofmeister series. Curr. Opin Chem. Biol. 2006, 10 (6), 658–663. 10.1016/j.cbpa.2006.09.020. 17035073

[ref107] Wang C.; Zeng X.; Qu J.; Cairney J. M.; Meng Q.; Cullen P. J.; Pei Z. Salt-tolerance training enabled flexible molten hydrate gel electrolytes for energy-dense and stable zinc storage. Matter 2023, 6 (11), 3993–4012. 10.1016/j.matt.2023.08.019.

[ref108] Wang S.; Urban M. W. Self-healing polymers. Nat. Rev. Mater. 2020, 5 (8), 562–583. 10.1038/s41578-020-0202-4.

[ref109] Zhang Z.; Gao Y.; Gao Y.; Jia F.; Gao G. A self-adhesive, self-healing zwitterionic hydrogel electrolyte for high-voltage zinc-ion hybrid supercapacitors. Chem. Eng. J. 2023, 452, 139014 10.1016/j.cej.2022.139014.

[ref110] Wang X.-H.; Song F.; Qian D.; He Y.-D.; Nie W.-C.; Wang X.-L.; Wang Y.-Z. Strong and tough fully physically crosslinked double network hydrogels with tunable mechanics and high self-healing performance. Chem. Eng. J. 2018, 349, 588–594. 10.1016/j.cej.2018.05.081.

[ref111] Xu T.; Yang D.; Zhang S.; Zhao T.; Zhang M.; Yu Z.-Z. Antifreezing and stretchable all-gel-state supercapacitor with enhanced capacitances established by graphene/PEDOT-polyvinyl alcohol hydrogel fibers with dual networks. Carbon 2021, 171, 201–210. 10.1016/j.carbon.2020.08.071.

[ref112] Pei Z.; Ding L.; Wang C.; Meng Q.; Yuan Z.; Zhou Z.; Zhao S.; Chen Y. Make it stereoscopic: interfacial design for full-temperature adaptive flexible zinc-air batteries. Energy Environ. Sci. 2021, 14 (9), 4926–4935. 10.1039/D1EE01244D.

[ref113] Yin J.; Wei K.; Zhang J.; Liu S.; Wang X.; Wang X.; Zhang Q.; Qin Z.; Jiao T. MXene-based film electrode and all-round hydrogel electrolyte for flexible all-solid supercapacitor with extremely low working temperature. Cell Rep. Phys. Sci. 2022, 3 (5), 100893 10.1016/j.xcrp.2022.100893.

[ref114] Sun W.; Yang J.; Ji X.; Jiang H.; Gai L.; Li X.; Liu L. Antifreezing zwitterionic hydrogel electrolyte with high conductivity at subzero temperature for flexible sensor and supercapacitor. Sust. Mater. Technol. 2022, 32, e00437 10.1016/j.susmat.2022.e00437.

[ref115] Yang Y.; Guan L.; Li X.; Gao Z.; Ren X.; Gao G. Conductive organohydrogels with ultrastretchability, antifreezing, self-healing, and adhesive properties for motion detection and signal transmission. ACS Appl. Mater. Interfaces 2019, 11 (3), 3428–3437. 10.1021/acsami.8b17440. 30592212

[ref116] Qin Z.; Sun X.; Zhang H.; Yu Q.; Wang X.; He S.; Yao F.; Li J. A transparent, ultrastretchable and fully recyclable gelatin organohydrogel based electronic sensor with broad operating temperature. J. Mater. Chem. A 2020, 8 (8), 4447–4456. 10.1039/C9TA13196E.

[ref117] Tao F.; Qin L.; Wang Z.; Pan Q. Self-Healable and Cold-Resistant Supercapacitor Based on a Multifunctional Hydrogel Electrolyte. ACS Appl. Mater. Interfaces 2017, 9 (18), 15541–15548. 10.1021/acsami.7b03223. 28421735

[ref118] Wang Z.; Liu J.; Zhang J.; Hao S.; Duan X.; Song H.; Zhang J. Novel chemically cross-linked chitosan-cellulose based ionogel with self-healability, high ionic conductivity, and high thermo-mechanical stability. Cellulose 2020, 27 (9), 5121–5133. 10.1007/s10570-020-03144-3.

[ref119] Zhu A.; Huang J.; Xie H.; Yue W.; Qin S.; Zhang F.; Xu Q. Use of a superbase/DMSO/CO2 solvent in order to incorporate cellulose into organic ionogel electrolyte for flexible supercapacitors. Chem. Eng. J. 2022, 446, 137032 10.1016/j.cej.2022.137032.

[ref120] Lu J.; Gu J.; Hu O.; Fu Y.; Ye D.; Zhang X.; Zheng Y.; Hou L.; Liu H.; Jiang X. Highly tough, freezing-tolerant, healable and thermoplastic starch/poly(vinyl alcohol) organohydrogels for flexible electronic devices. J. Mater. Chem. A 2021, 9 (34), 18406–18420. 10.1039/D1TA04336F.

[ref121] Hu M.; Wang J.; Liu J.; Wang P.; Feng Y.; Wang H.; Nie N.; Wang Y.; Huang Y. A flour-based one-stop supercapacitor with intrinsic self-healability and stretchability after self-healing and biodegradability. Energy Storage Mater. 2019, 21, 174–179. 10.1016/j.ensm.2018.12.013.

[ref122] Wang R.; Yao M.; Huang S.; Tian J.; Niu Z. Sustainable Dough-Based Gel Electrolytes for Aqueous Energy Storage Devices. Adv. Funct. Mater. 2021, 31 (14), 2009209 10.1002/adfm.202009209.

[ref123] Durukan M. B.; Keskin D.; Tufan Y.; Dincer O.; Cicek M. O.; Yildiz B.; Çınar Aygün S.; Ercan B.; Unalan H. E. An Edible Supercapacitor Based on Zwitterionic Soy Sauce-Based Gel Electrolyte. Adv. Funct. Mater. 2024, 34, 2307051 10.1002/adfm.202307051.

[ref124] Didwal P. N.; Verma R.; Nguyen A. G.; Ramasamy H. V.; Lee G. H.; Park C. J. Improving Cyclability of All-Solid-State Batteries via Stabilized Electrolyte-Electrode Interface with Additive in Poly(propylene carbonate) Based Solid Electrolyte. Adv. Sci. 2022, 9 (13), 2105448 10.1002/advs.202105448. PMC9069196 35240003

[ref125] Bi W.; Wang J.; Jahrman E. P.; Seidler G. T.; Gao G.; Wu G.; Cao G. Interface Engineering V(2) O(5) Nanofibers for High-Energy and Durable Supercapacitors. Small 2019, 15 (31), e1901747 10.1002/smll.201901747. 31215181

[ref126] Huang C.; Zhang J.; Snaith H. J.; Grant P. S. Engineering the Membrane/Electrode Interface To Improve the Performance of Solid-State Supercapacitors. ACS Appl. Mater. Interfaces 2016, 8 (32), 20756–20765. 10.1021/acsami.6b05789. 27467593

[ref127] Wang H.; Wu J.; Qiu J.; Zhang K.; Shao J.; Yan L. In situ formation of a renewable cellulose hydrogel electrolyte for high-performance flexible all-solid-state asymmetric supercapacitors. Sustainable Energy & Fuels 2019, 3 (11), 3109–3115. 10.1039/C9SE00339H.

[ref128] Fang L.; Cai Z.; Ding Z.; Chen T.; Zhang J.; Chen F.; Shen J.; Chen F.; Li R.; Zhou X.; et al. Skin-Inspired Surface-Microstructured Tough Hydrogel Electrolytes for Stretchable Supercapacitors. ACS Appl. Mater. Interfaces 2019, 11 (24), 21895–21903. 10.1021/acsami.9b03410. 31124644

[ref129] Liang Y.; Song Q.; Chen Y.; Hu C.; Zhang S. Stretch-Induced Robust Intrinsic Antibacterial Thermoplastic Gelatin Organohydrogel for a Thermoenhanced Supercapacitor and Mono-gauge-factor Sensor. ACS Appl. Mater. Interfaces 2023, 15 (16), 20278–20293. 10.1021/acsami.3c02255. 37043180

[ref130] Sun L.; Yao Y.; Dai L.; Jiao M.; Ding B.; Yu Q.; Tang J.; Liu B. Sustainable and high-performance Zn dual-ion batteries with a hydrogel-based water-in-salt electrolyte. Energy Storage Mater. 2022, 47, 187–194. 10.1016/j.ensm.2022.02.012.

[ref131] Park J.; Lee J.; Kim W. Redox-Active Water-in-Salt Electrolyte for High-Energy-Density Supercapacitors. ACS Energy Lett. 2022, 7 (4), 1266–1273. 10.1021/acsenergylett.2c00015.

[ref132] Dou Q.; Lu Y.; Su L.; Zhang X.; Lei S.; Bu X.; Liu L.; Xiao D.; Chen J.; Shi S.; et al. A sodium perchlorate-based hybrid electrolyte with high salt-to-water molar ratio for safe 2.5 V carbon-based supercapacitor. Energy Storage Mater. 2019, 23, 603–609. 10.1016/j.ensm.2019.03.016.

[ref133] Xiao D.; Dou Q.; Zhang L.; Ma Y.; Shi S.; Lei S.; Yu H.; Yan X. Optimization of Organic/Water Hybrid Electrolytes for High-Rate Carbon-Based Supercapacitor. Adv. Funct. Mater. 2019, 29 (42), 1904136 10.1002/adfm.201904136.

[ref134] Neto C.; Pham H. T.; Omnée R.; Canizarès A.; Slodczyk A.; Deschamps M.; Raymundo-Piñero E. Exploring the Carbon/Electrolyte Interface in Supercapacitors Operating in Highly Concentrated Aqueous Electrolytes. ACS Appl. Mater. Interfaces 2022, 14 (39), 44405–44418. 10.1021/acsami.2c12010. 36150165

[ref135] Przypis M.; Wawoczny A.; Gillner D. Biomass and Cellulose Dissolution—The Important Issue in Renewable Materials Treatment. Appl. Sci. 2023, 13 (2), 1055 10.3390/app13021055.

[ref136] Salanne M.; Rotenberg B.; Naoi K.; Kaneko K.; Taberna P. L.; Grey C. P.; Dunn B.; Simon P. Efficient storage mechanisms for building better supercapacitors. Nat. Energy 2016, 1, 16070 10.1038/nenergy.2016.70.

